# Virtual Exposure With Response Prevention for Obsessive-Compulsive Disorder: Randomized Controlled Trial

**DOI:** 10.2196/79326

**Published:** 2026-05-27

**Authors:** Lara Rolvien, Lena Jelinek, Luzie Lohse, Steffen Moritz, Lisa Borgmann, Jürgen Gallinat, Simone Kühn, Franziska Miegel

**Affiliations:** 1 Department of Psychiatry and Psychotherapy, University Medical Center Hamburg-Eppendorf, Martinistr 52, Hamburg, 20246, Germany, 49 040741055868; 2 Center for Environmental Neuroscience, Max Planck Institute for Human Development, Berlin, Germany

**Keywords:** virtual reality, exposure with response prevention, obsessive-compulsive disorder, contamination, checking

## Abstract

**Background:**

Despite being highly effective and guideline-recommended for obsessive-compulsive disorder, exposure with response prevention is often underused due to barriers from both therapists and patients. Virtual reality may help address these barriers.

**Objective:**

This study aimed to assess the efficacy and acceptance of virtual exposure with response prevention (VERP) in patients with contamination obsessive-compulsive disorder (conOCD) and checking obsessive-compulsive disorder (checkOCD) and to identify factors influencing treatment success.

**Methods:**

Within a randomized controlled trial, 80 participants (40 with conOCD and 40 with checkOCD) were allocated to an intervention group, which received outpatient care as usual (CAU) supplemented by 6 weekly VERP sessions (VERP group), or to a control group receiving CAU alone (CAU group). Assessments occurred at baseline (t0), postintervention (t1), and at 3-month follow-up (t2). The reduction in obsessive-compulsive symptoms, as measured with the Yale-Brown Obsessive-Compulsive Scale, served as the primary outcome.

**Results:**

Overall, obsessive-compulsive symptoms showed no greater improvement in the VERP group compared to the CAU group from t0 to t1 or t2 (η_p_^2^=0.003-0.014). In exploratory subsample analyses, we observed a trend toward an interaction effect, showing greater reduction in obsessions among participants with checkOCD from t0 to t1 (*F*_1,52_=3.91; *P*=.05; η_p_^2^=0.070) and greater anxiety reduction among participants with conOCD from t0 to t2 (*F*_1,45_=4.11; *P*=.05; η_p_^2^=0.084). Exploratory moderation analysis suggested that adding VERP to CAU was more effective for those in the VERP group who had not prematurely discontinued prior psychological treatment and those with checking behavior. Sense of presence was only moderate, unrelated to outcome, and patient satisfaction with VERP was positive overall.

**Conclusions:**

While overall symptom reduction did not significantly differ between groups, exploratory analyses suggested trend-level differences in treatment response across obsessive-compulsive disorder subtypes and patient characteristics. These findings should be interpreted cautiously and are best viewed as hypothesis-generating.

## Introduction

### Background

Obsessive-compulsive disorder (OCD) is a common chronic and disabling condition characterized by recurrent intrusive, distress-provoking thoughts, images, ideas, and impulses (obsessions) as well as repetitive overt or covert rituals (compulsions) aimed at reducing distress and unpleasant emotions such as anxiety or disgust. Obsessions and compulsions can burden and disrupt daily life, which highlights the negative impact on patients’ quality of life [1]. With lifetime prevalence estimates of OCD ranging from 1.3% to 2.5% in recent meta-analyses [2,3] and OCD’s ranking as one of the 10 leading causes of disability worldwide due to its chronic nature [4], the need for appropriate treatment is evident.

Cognitive behavioral therapy (CBT) that includes exposure with response prevention (ERP) is the recommended first-line treatment for OCD [5-7]. In ERP, patients are encouraged to face their fears stemming from their obsessions (exposure) and are taught to resist the urge to engage in compulsive behaviors (response prevention). Classic ERP can take various forms, with the most common being in vivo exposure in real-life scenarios. ERP in imagined scenarios is called in sensu, with the latter yielding lower long-term success rates [8]. The idea behind ERP is to help patients realize that the feared stimuli are not as threatening as perceived and that their distress, anxiety, or other unpleasant emotions naturally decrease (or can be managed) without the need for compulsive behaviors, ultimately leading to changes in maladaptive cognitive processes [5,9,10].

Despite its well-established effectiveness in treating OCD [11], ERP is not used to its full potential. Several patient- and therapist-related factors may be responsible for this [12]. For the therapist, ERP can be time-intensive and may pose practical challenges, including the need for longer or more frequent sessions and the coordination of in vivo exposures [13,14]. Moreover, therapists have reported that the exposure is stressful for them, they do not feel safe conducting it, they are afraid of patients’ reactions, and they fear harming the patient [15]. According to Maltby and Tolin [16], even among therapists who identify themselves as adhering to a cognitive behavioral approach, the implementation of evidence-based therapies such as ERP remains insufficient, with use rates remaining below 50%. As for the patients, they often fear exposure, lack motivation, prefer self-help exposure, and/or perceive logistical challenges [14]. It has been estimated that around 25% of patients with OCD refuse ERP [16]. Together, the barriers on the patients’ and the therapists’ sides may result in what Moritz et al [14] refer to as “phobie à deux,” hindering successful treatment and symptom improvement in the patient.

### Exposure Therapy in Virtual Reality

Exposure therapy in virtual reality (VRET) is a promising new treatment strategy with the potential to overcome some of the described treatment barriers, particularly logistical challenges and time difficulties for therapists as well as patient fears. Virtual reality (VR) is a computer-generated simulation, enabling real-time interaction with the environment. It offers a nurturing and safe environment for patients, fostering increased self-efficacy [17]. Consequently, VRET is often considered a promising or advantageous alternative compared to in vivo or in sensu exposure in certain contexts, particularly due to its potential to increase patient acceptance and reduce refusal rates, rather than being universally superior [18,19]. Leveraging VR within psychotherapy offers significant benefits, including the logistical advantages of tailoring environments and scenarios, controlling the exposure process, and conducting therapy sessions in the therapist’s room [20]. By using VR, therapists can tailor the virtual environment to match the specific fears of each patient, subsequently offering guidance and support while monitoring their progress within the virtual environment [21]. Moreover, the interactivity of VR allows for standardized, controlled, and replicable exposure environments, which can improve the therapeutic process, by ensuring consistent delivery of exposure tasks, reducing reliance on patient imagination or therapist improvisation, and facilitating the safe practice of challenging situations. These features may lower barriers to treatment access and enhance efficiency [17,18].

VRET for anxiety disorders has been well studied and has shown efficacy for specific phobias, social anxiety disorder, posttraumatic stress disorder, and panic disorder, as a meta-analysis of 30 studies on VRET revealed [22]. However, the success of VRET in this meta-analysis was similar to in vivo exposure. In more severe anxiety disorders and posttraumatic stress disorder, according to another meta-analysis [23], VRET’s superiority to CBT with ERP was small and not significant.

### Virtual Exposure With Response Prevention for OCD

The inherent challenges posed by the nature of OCD make the use of VR in patients with OCD more difficult; it is less researched and less disseminated in clinical practice despite its possible advantages for treatment. So far, it is unclear whether the treatment effects for anxiety disorders generalize to OCD, which is characterized by high idiosyncrasy and cognitive avoidance, especially during in sensu exposure [24]. Moreover, the stimuli in OCD are more heterogeneous than in most anxiety disorders, making it difficult to cover all comprehensively in one virtual environment. For patients, particularly those afflicted with contamination obsessive-compulsive disorder (conOCD), the use of a shared device, such as VR glasses, may pose considerable challenges due to concerns regarding potential contamination by previous users [25].

However, preliminary research has shown that virtual exposure with response prevention (VERP) can be a valuable tool in the treatment of OCD. Studies have found that VERP can effectively provoke anxiety, disgust, and checking in patients with OCD [26-28], and patients have reported a good sense of “presence” (ie, the feeling of being there in a virtual environment) and increased anxiety (standardized mean difference=2.92; 95% CI 1.89‐3.94; *P*<.001; *I*^2^=95%) and disgust (standardized mean difference=2.52; 95% CI 1.36‐3.68; *P*<.001; *I*^2^=95%) levels in response to VERP scenarios compared to healthy controls in a meta-analysis of 11 primary studies [27]. Furthermore, a trend correlation between anxiety level and emotional engagement (*r*=0.87; *P*≥.05) as well as anxiety and sense of presence (*r*=0.88; *P*≥.05) during VERP has been found in a primary study including 4 women with OCD [29]. Laforest et al [24] investigated VERP in 3 patients over the course of 12 weeks and found a significant reduction in obsessions and compulsions in all patients, as assessed by visually inspecting the graphs of the daily assessments and an autoregressive moving average analysis, although the effects were not maintained in 2 of the 3 patients after 12 months. In addition, VERP has been found to be effective in managing obsessive-compulsive (OC) symptoms, particularly when traditional ERP has been unsuccessful, as reported in a single case report [30]. A recent case series that included 8 patients with contamination-related OCD revealed that VERP was able to induce distress (subjectively measured, *d*=1.11) and arousal (assessed with Galvanic Skin Response: *d*=4.84 and heart rate: *d*=2.38) associated with disgust at large effect sizes. VERP also evoked a moderate sense of presence but found a low rate of symptom reduction as measured with the Yale-Brown Obsessive-Compulsive Scale (Y-BOCS) total scale (*t*_7_=2.30; *P*=.06; *d*=1.82 [25]). In another study, 21 nonclinical participants with contamination fears either received VERP or were assigned to a control group, resulting in reduced anxiety (*F*_1,19_=11.75; *P*<.01; η_p_^2^=0.38), disgust (*F*_1,19_=18.53; *P*<.001; η_p_^2^=0.49), and urge to wash hands (*F*_1,19_=17.03; *P*<.01; η_p_^2^=0.47) in the experimental group [31]. In line with these findings, Javaherinenani et al [21] found that CBT with VERP significantly reduced the severity of OC symptoms in the intervention group undergoing a 12-week treatment compared to a conventional CBT treatment including ERP components (Y-BOCS total score: *F*=60.97; *P*<.001; η_p_^2^=0.82).

Although there are initial indications that VERP can be effectively used in the treatment of OCD, larger-scale research is needed to determine its full potential, especially when compared to care as usual (CAU; defined to allow all outpatient treatments to be used, such as CBT with ERP). A recent systematic review evaluating the evidence for the use of VR in both the diagnosis and treatment of mental disorders found that the smallest number of studies focused on OCD [32]. This review is not the only one to conclude that the research gap in VERP for OCD is marked and that further studies are urgently needed. Beyond establishing efficacy, it is also important to evaluate patients’ satisfaction with the treatment [25] as well as their sense of presence—a factor known to elicit anxiety in VRET and thus one that may influence treatment effectiveness [29,33].

In this study, we focused specifically on the symptom dimensions of conOCD and checking obsessive-compulsive disorder (checkOCD). These 2 domains are among the most prevalent and clinically relevant manifestations of OCD, consistently identified in factor-analytic studies and frequently associated with substantial distress and impairment [34-36]. They also represent prototypical forms of compulsive behavior that are well-suited to examine the feasibility and potential effects of novel interventions such as VERP. Importantly, although many individuals with OCD experience symptoms across multiple domains, classification into patients with conOCD or patients with checkOCD in our trial was based on the predominant symptom dimension, as determined through clinical interview and supported by self-report. This approach reflects the conceptual and empirical relevance of symptom dimensions while acknowledging the dimensional nature of OCD presentations.

### Study Objectives

The primary objective of this study was to examine the incremental benefit of adding VERP to CAU in patients with OCD, compared to CAU alone. Specifically, we aimed to test whether VERP plus CAU would lead to greater reductions in overall OCD severity (measured by the Y-BOCS) than CAU alone. In addition, we investigated potential differences between conOCD and checkOCD, hypothesizing that patients with conOCD would benefit more strongly from VERP than patients with checkOCD due to the higher ecological validity of contamination-related VR scenarios. Finally, we explored patient satisfaction with and sense of presence in VERP and examined the potential relationship between these factors and efficacy.

## Methods

### Design

In the framework of a randomized controlled trial (see Checklist 1 for the CONSORT list[Consolidated Standards of Reporting Trials]), patients with conOCD and patients with checkOCD were randomly assigned to either the VERP or the CAU group after baseline assessment (t0). After 6 weeks, all patients were invited to a second assessment (post assessment, t1). After 3 more months, an online survey and a telephone interview were conducted (follow-up, t2). The principal investigators (LR and FM) carried out the randomization, which was based on a randomization plan (1:1 allocation rule) that was conducted by an online randomization program. Additionally, randomization was stratified by type of compulsions (contamination or checking), with half of the patients with conOCD being allocated to the intervention group and half to the CAU group, and the same procedure applied to patients with checkOCD. Patients were informed about the group assignment by a sequentially numbered envelope they received after t0. Before leaving the room after the t0 assessment, the raters, who were blind to the patients’ group assignments, reminded the patients not to reveal their group assignment at the following assessments. Additionally, patients in the intervention group as well as the CAU group were permitted to continue their routine medical care (such as CBT or psychiatric medication), which was carefully recorded.

### Ethical Considerations

The study was conducted in accordance with the Declaration of Helsinki, received ethics approval (local ethics committee of the clinic: LPEK-0020), and was preregistered with the German Clinical Trials Register (DRKS00016929). All participants provided written informed consent prior to participation. Informed consent procedures also included permission for the use of anonymized study data for analysis and publication. No additional consent was required for the present analyses. All data were pseudonymized at the point of collection and stored on secure servers in accordance with data protection regulations. No personal identifiers were retained, and confidentiality of participants’ information was ensured throughout the study. No financial compensation was offered for study participation, but, as an incentive, after the last assessment, patients in the CAU group received a self-help manual for OCD—My Metacognitive Training [37]. No identifying images or other personal details of study participants are included in this paper.

### Participants and Procedure

The recruitment strategies included a Google AdWords campaign, promotion in related forums and posts on websites, posters, describing the study to the anxiety outpatient ward of the clinic and local therapists, as well as getting in touch with previous study participants who had given permission to be contacted again for future studies. A telephone interview conducted by the principal investigators (LR, FM, and LL) screened interested individuals for eligibility. In this screening, interested individuals were asked whether they predominantly experienced washing and/or checking compulsions. This inclusion criterion was reassessed during the baseline interview (t0) using the Y-BOCS symptom checklist to ensure accurate identification of each participant’s predominant symptom dimension. Participants who reported both types of compulsions were further asked to identify which behavior was more dominant, distressing, and restrictive. Based on this self-assessment, participants were classified as either having conOCD or checkOCD. It is important to note that overlap of compulsive behaviors is common, and the classification reflects the symptom domain most prominently impacting the individual, while other OCD symptoms may also be present to a lesser extent. Inclusion and exclusion criteria are presented in Textbox 1. If eligible, patients were invited to an in-person t0 assessment and a t1 assessment 6 weeks later that were conducted by trained interviewers, and they received a link to an online assessment containing the questionnaires (secondary outcome measures). After the t0 assessment, patients in the intervention group participated in VERP sessions for 6 weeks, with weekly sessions lasting 60 to 90 minutes each. The t2 assessment 3 months after t1 was carried out online and via telephone.

Textbox 1. **Inclusion and exclusion criteria.**

**Inclusion criteria**

- Predominant presence of contamination obsessive-compulsive disorder or checking obsessive-compulsive disorder
- Provision of written informed consent
- Age between 18 and 75 years
- Sufficient command of the German language
- Willingness to participate in virtual exposure with response prevention for 6 weeks

**Exclusion criteria**

- Lifetime symptoms of psychosis or mania
- Severe neurological disorder
- Acute suicidality

### Treatment

All 6 treatment sessions (1 session per week) were highly structured and used a treatment manual (based on a CBT manual for OCD by Oelkers and Hautzinger [38]). The first 2 sessions prepared the patient for the VERP sessions (symptom anamnesis, psychoeducation on ERP, presentation of a video of the VR environment, and rating of the OC-triggering stimuli to design the individual ERP). In the third session, the patient was familiarized with the VR equipment through testing the VR devices in a neutral VR setting (ie, a neutral, unfurnished virtual room). After that, the therapist guided the patient through the actual VERP session (eg, by encouraging them to look at and engage with the stimuli). In sessions 4 to 6, further VERPs were conducted, starting at medium intensity in session 3 and increasing the intensity throughout the following sessions. At the beginning of sessions 3 to 6, expectations on what would happen during VERP were identified beforehand and discussed afterward (eg, by noting that the feared consequence, such as fainting, had not occurred, representing an expectancy violation [39]). To minimize the occurrence of mental checking during VERP sessions, patients were explicitly informed about the concept of “mental checking” and instructed to refrain from engaging in it. Therapists provided detailed guidance on how to behave following each exposure session, including instructions not to wash hands, use disinfectants, or change clothes immediately. These measures were implemented to ensure response prevention and to reduce the likelihood that participants classified as having checkOCD would engage in covert checking or that patients with conOCD would perform their compulsive rituals immediately after the session. Therapists were psychotherapists in CBT training or licensed psychotherapists supervised by an experienced CBT psychotherapist (LJ).

**Figure 1.**
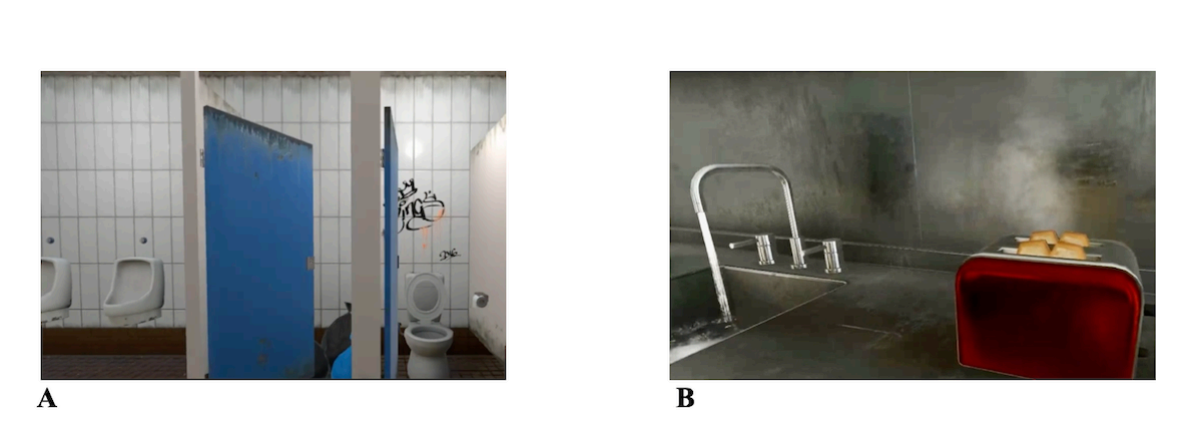
(A) Excerpt of the VR environment for patients with washing compulsions. (B). Excerpt of the VR environment for patients with checking compulsions. VR: virtual reality.

An HTC Vive Pro headset was used to conduct the VERP. The VR environments were conducted by Unity (version 2018.2.9f1). In the virtual environment, patients moved by using controllers that teleported (StreamVR Teleport) them to the intended location. During VERP, therapists were able to guide patients because they could see on a computer screen what the patient was seeing. Two different virtual environments were available: 1 public restroom addressing conOCD (Figure 1A) and 1 apartment with various technical and other checking-related devices addressing checkOCD (Figure 1B). The public restroom included, among other things, a dirty sink, urine, feces, blood, and a wet floor. In this environment, patients were also instructed to interact directly with the stimuli, for example, by touching the “dirty” floor or sitting on a “dirty toilet” (represented by a regular chair in the real room). Participants were instructed not to wash or disinfect their hands, body, or clothes afterward, ensuring response prevention. In the apartment, participants were confronted with various electrical devices, candles, and water taps. To ensure response prevention in this environment, participants here were explicitly instructed to switch on relevant devices (eg, stove or microwave and water tap) at the beginning of the exercise, then switch them off only once, and immediately leave the room and subsequently the apartment. The front door could be closed with a key, and repeated checking was not permitted.

### Control Condition

In this study, a CAU control group was included. CAU was defined so that all outpatient treatments could be used, including CBT with ERP. It was assessed whether participants had received outpatient psychotherapy in general during the intervention period, and additionally, whether they had received guided ERP in vivo or unguided ERP in vivo.

### Measures

At baseline (t0), sociodemographic and clinical variables were assessed through a structured interview. The following information was collected: gender (women or men), age (in years), and qualification for university study. In addition, treatment-related variables were recorded, including current medication (antidepressant, combination, other, and none), psychotherapy experience (current, in the past, current and in the past, or discontinued prematurely), illness duration (in years), and age at onset (in years). No monitoring, management, or reassessment of medication occurred during the study, as all participants were outpatients. All measures used in the study are summarized in Table 1.

**Table 1.** Measures used in the study.

Title	Abbreviation	Content and psychometrics	Assessment times
Primary outcome			
Yale-Brown Obsessive-Compulsive Scale	Y-BOCS	The Y-BOCS [40,41] is a half-structured interview assessing OC^a^ symptoms by a symptom checklist and a severity rating (including 19 items). Psychometric properties of the German version have been shown to be good to very good [42].	t0, t1, and t2
Secondary outcomes			
Obsessive-Compulsive Inventory—Revised	OCI-R	The OCI-R [43,44] is a self-rated questionnaire capturing OC symptoms. The German version of the OCI-R showed good psychometric properties [44].	t0, t1, and t2
Beck Depression Inventory-II	BDI-II	The BDI-II [45] is a widely used 21-item self-report questionnaire measuring the severity of depression symptomatology in adolescents and adults. The German version of the BDI-II has been shown to have good reliability and validity in both clinical and nonclinical populations [46].	t0, t1, and t2
Generalized Anxiety Disorder Scale-7	GAD-7	The GAD-7 [47] is a 7-item questionnaire that aims to detect the severity of clinical symptoms of generalized anxiety disorder. The scale has shown good psychometric properties in a German sample and can therefore be considered a reliable scale [48].	t0, t1, and t2
Obsessive Beliefs Questionnaire-44	OBQ-44	The OBQ-44 [49,50] evaluates belief domains pertinent to OCD^b^, including control of thoughts, importance of thoughts, responsibility, intolerance of uncertainty, overestimation of threat, and perfectionism. The questionnaire has shown very good psychometric properties [51].	t0, t1, and t2
World Health Organization Quality of Life—BREF	WHOQOL-BREF	Quality of life was assessed with the global item of the WHOQOL-BREF, which served as a brief indicator of participants’ overall quality of life while minimizing assessment burden [52].	t0, t1, and t2
Subjective Appraisal Rating	—^c^	The questionnaire capturing the subjective appraisal of the VERP^d^ (only by patients in the VERP group) was based on ones used by Jelinek et al [53,54] and was adapted to fit the assessment of VERP. One open question asks patients for their appraisal of the VERP and leaves space for a personal comment.	t1
Igroup Presence Questionnaire	IPQ	The IPQ [55] records the degree patients feel being (or feeling present) in the virtual environment. The scale consists of three subscales assessing (1) spatial presence, (2) involvement, and (3) experienced realism. One additional item assesses the general sense of presence. Psychometric properties of the IPQ have been reported to be good [55].	t1
Mini-International Neuropsychiatric Interview	MINI	Psychiatric diagnoses were assessed using the MINI [56], a structured diagnostic interview designed to identify major axis I psychiatric disorders according to DSM-IV^e^ and ICD-10^f^ criteria. The MINI was administered by a trained interviewer to confirm eligibility and to document comorbid psychiatric conditions.	t0

a OC: obsessive-compulsive.

b OCD: obsessive-compulsive disorder.

c Not available.

d VERP: virtual exposure with response prevention.

e DSM-IV: Diagnostic and Statistical Manual of Mental Disorders IV.

f ICD-10: International Classification of Diseases, 10th Revision.

### Power Analysis

As Carl et al [22] reported medium to large effect sizes in their meta-analysis on VRET in anxiety disorders, we expected similar effect sizes for this study. Thus, to detect an effect size of *d*=0.80 (α=.05) with a power of 0.80, a sample size of 70 participants was needed according to G*Power [57]. ERP often has a high dropout rate [58], but we expected it to be lower in this study due to the less frightening nature of the operationalization by VR; thus, we expected a dropout rate of 15%. This led us to recruit a total sample of 80 participants.

### Strategy of Data Analysis

A complete case (CC) analysis (patients who provided data at all assessments) and an intention-to-treat (ITT) analysis (patients who provided only t0 data) were conducted. Analyses of covariance (ANCOVAs) were conducted with group (VERP group vs CAU group) as the between-subject factor, the t0 scores of the respective outcome as the covariates, and change scores as the dependent variables. Change scores were defined as the difference between baseline and posttreatment (t0–t1) for the Y-BOCS, Obsessive-Compulsive Inventory—Revised (OCI-R), Generalized Anxiety Disorder Scale-7 (GAD-7), Beck Depression Inventory-II (BDI-II), Obsessive Beliefs Questionnaire-44 (OBQ-44), and World Health Organization Quality of Life—BREF (WHOQOL-BREF), and as the difference between baseline and follow-up (t0–t2) for the same measures. Exploratory analyses included the Y-BOCS subscales (obsessions and compulsions). These analyses are reported as exploratory due to the high interrelatedness of the outcomes and the risk of type I error; results should therefore be interpreted cautiously. The imputation of missing values for the ITT analysis was performed by multiple imputation. Imputations were performed using fully conditional specification with predictive mean matching (20 nearest neighbors), generating 100 imputed datasets. The imputation model included all primary and secondary outcome change scores (Y-BOCS total and subscales, Obsessive Compulsive Inventory, Beck Depression Inventory, GAD-7, Obsessive Beliefs Questionnaire, and quality of life), as well as relevant baseline covariates (age, gender, marital status, duration of illness, age at onset, and number of previous outpatient treatments). Pooled estimates were obtained across imputations according to Rubin’s rules. Within-group differences on the Y-BOCS (and for exploratory purposes on its subscales), OCI-R, GAD-7, BDI-II, OBQ-44, and WHOQOL-BREF were analyzed with paired-sample 2-tailed *t* tests. Pearson correlations were analyzed for the relationship between sense of presence (measured with the Igroup Presence Questionnaire [IPQ]) and reduction in the primary outcome (Y-BOCS). For exploratory subsample analyses addressing the 2 types of OCD (conOCD and checkOCD), a 2×2 ANCOVA that included type of OCD as an additional fixed factor was conducted for both t0-t1 and t0-t2 for the Y-BOCS (and for exploratory purposes for its subscales), OCI-R, GAD-7, BDI-II, OBQ-44, and WHOQOL-BREF. Here, only trend or significant effects are reported for clarity and conciseness. The assumption of homogeneity of variances was tested with the Levene test. An exploratory moderation analysis was conducted using the SPSS macro PROCESS [59] for the CC sample for hypothesis-generating purposes. Here, the difference in the primary outcome (Y-BOCS total score) from t0-t1 and t0-t2 served as the dependent variable and group as the independent variable. Baseline sociodemographic variables as well as total and subscales of all primary and secondary measures assessed at t0 were included as possible moderators (only significant or trend interaction effects are reported). Patient satisfaction was analyzed descriptively by displaying positively (combined completely agree and agree) and negatively formulated (combined disagree and completely disagree) separately.

## Results

### Overview

**Figure 2.**
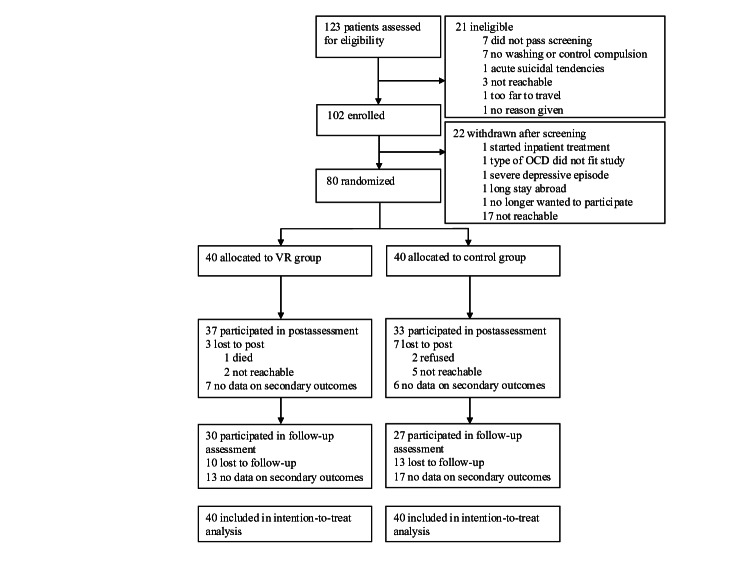
Study flowchart. OCD: obsessive-compulsive disorder; VR: virtual reality.

A total of 123 patients were screened for eligibility (see Figure 2 for details). The final sample consisted of 80 patients with OCD (40 patients with conOCD and 40 with checkOCD). At baseline, 1 participant per group did not complete the secondary outcome measures, resulting in a baseline sample size of 39 per group for these variables, while the full ITT sample (n=40 per group) was retained for all analyses. In total, 59 (73.8%) participants were women, and the mean age of the total sample was 36.85 (SD 11.44) years. The mean OCD duration was 18.04 (SD 14.22) years, and on average, the participants were aged 19.14 (SD 10.67) years at the onset of their illness.

The VERP and CAU groups did not differ in sociodemographics, treatment variables, or psychopathology at t0 (Table 2). The sample showed severe OC symptoms (Y-BOCS) and moderate depressive symptoms (BDI-II) and anxiety symptoms (GAD-7). According to the Mini-International Neuropsychiatric Interview [56], main comorbidities were depression (53/80, 66.2%, with 18/80, 22.5% having a current depressive episode and 34/80, 42.5% a past depressive episode) and anxiety (current or lifetime panic disorder: 12/80, 15%; generalized anxiety disorder: 10/80, 12.5%; agoraphobia: 5/80, 6.3%; social phobia: 3/80, 3.8%). A majority of the sample had previously undergone psychotherapy (69/80, 86.2%), of which 14 of 80 (17.5%) had done so only currently, 35 of 80 (43.8%) only in the past, and 20 of 80 (25%) both currently and in the past. In total, 26 of 80 (32.5%) patients had prematurely discontinued a treatment in the past.

**Table 2.** Baseline characteristics with psychopathology (intention-to-treat sample) and treatment use during the intervention period (reported at post)^a^.

	VERP^b^ group (n=40)	CAU^c^ group (n=40)	Statistics	*P* value
Demographic characteristics				
Gender, n (%)			*χ*^2^_1_=0.6	.45
Women	28 (70%)	31 (77.5%)		
Men	12 (30%)	9 (22.5%)		
Age (years), mean (SD)	38.18 (11.61)	35.53 (11.26)	*t*_78_=1.04	.15
Qualified for university study	32 (80%)	29 (72.5%)	*χ*^2^_2_=0.7	.71
Treatment variables				
Medication, n (%)			*χ*^2^_4_=5.3	.26
Antidepressant	15 (37.5%)	14 (35%)		
Combination	4 (10%)	5 (12.5%)		
Other	6 (15%)	1 (2.5%)		
None	15 (37.5%)	20 (50%)		
Psychotherapy experience, n (%)			*χ*^2^_3_=2.8	.43
Current	6 (15%)	8 (20%)		
In the past	17 (42.5%)	18 (45%)		
Current and in the past	9 (22.5%)	11 (27.5%)		
None	8 (20%)	3 (7.5%)		
Discontinued a previous treatment prematurely, n (%)	15 (37.5%)	11 (27.5%)	*χ*^2^_1_=1.1	.30
Illness duration (years), mean (SD)	18.23 (13.57)	17.86 (15.02)	*t*_78_=0.11	.46
Age at onset (years), mean (SD)	18.60 (11.58)	19.68 (9.80)	*t*_78_=–0.45	.33
Psychopathology, mean (SD)				
Y-BOCS^d^ total	24.40 (6.33)	24.25 (5.53)	*t*_78_=0.11	.46
Y-BOCS obsessions	11.78 (3.33)	11.60 (3.10)	*t*_78_=0.24	.40
Y-BOCS compulsions	12.63 (3.29)	12.65 (3.05)	*t*_78_=–0.04	.49
OCI-R^e^ total	31.77 (12.07)	31.18 (10.37)	*t*_76_=0.23	.41
OCI-R washing	7.16 (4.30)	6.69 (4.24)	*t*_76_=–0.48	.32
OCI-R obsessions	6.05 (3.21)	5.74 (3.08)	*t*_76_=–0.43	.33
OCI-R hoarding	3.54 (3.33)	3.49 (2.59)	*t*_76_=–0.08	.47
OCI-R ordering	4.54 (3.47)	4.72 (3.14)	*t*_76_=0.24	.41
OCI-R checking	7.05 (3.64)	7.44 (3.07)	*t*_76_=0.50	.31
OCI-R neutralizing	3.44 (3.72)	3.10 (3.39)	*t*_76_=–0.41	.34
BDI-II^f^	19.69 (11.83)	21.64 (11.74)	*t*_76_=–0.73	.23
GAD-7^g^	11.31 (5.07)	11.46 (4.47)	*t*_76_=–0.14	.44
OBQ-44^h^	188.54 (45.96)	191.44 (53.68)	*t*_76_=–0.26	.40
QoL^i^	2.90 (0.88)	2.82 (1.00)	*t*_76_=0.36	.36
Treatment use during the intervention period^j^				
Outpatient psychotherapy	17 (63%)	13 (56.5%)	*χ*^2^_1_=0.2	.64
Guided ERP^k^ in vivo	8 (29.6%)	4 (17.4%)	*χ*^2^_1_=1.0	.31
Unguided ERP in vivo	12 (44.4%)	7 (30.4%)	*χ*^2^_1_=1.0	.31

a For psychopathological questionnaires that were collected online (OCI-R, BDI-II, GAD-7, OBQ-44, and QoL), data are only available for 39 per group due to missing baseline responses from 1 participant in each group. All participants were retained in the intention-to-treat sample.

b VERP: virtual exposure with response prevention.

c CAU: care as usual.

d Y-BOCS: Yale-Brown Obsessive-Compulsive Scale.

e OCI-R: Obsessive-Compulsive Inventory—Revised.

f BDI-II: Beck Depression Inventory-II.

g GAD-7: Generalized Anxiety Disorder Scale-7.

h OBQ-44: Obsessive Beliefs Questionnaire-44.

i Global item of the World Health Organization Quality of Life—BREF (quality of life).

j VERP group: n=27; CAU group: n=23.

k ERP: exposure with response prevention.

At t1, 17 (63%) participants of the VERP group reported having received outpatient psychotherapy during the intervention period, with 8 (29.6%) participants having received therapist-guided ERP in vivo, and 12 (44.4%) having received unguided ERP in vivo (Table 2). In contrast, 13 (56.5%) participants in the CAU group reported having received outpatient psychotherapy during the intervention period, with 4 (17.4%) participants having received therapist-guided ERP in vivo, and 7 (30.4%) having received unguided ERP in vivo. Participation in outpatient therapy, guided or unguided ERP, did not significantly differ between the 2 groups (all *P*>.05). Regarding the differentiation according to OCD subtype (conOCD and checkOCD), 13 (56.5%) patients with conOCD reported having received outpatient psychotherapy, with 5 (21.7%) participants having received therapist-guided ERP in vivo and 10 (43.5%) unguided ERP in vivo. In contrast, 17 (63%) patients with checkOCD reported having received outpatient psychotherapy, with 7 (25.9%) participants having received therapist-guided ERP in vivo and 9 (33.3%) unguided ERP in vivo. Again, these differences did not reach statistical significance (all *P*>.05).

### Completion

The ITT sample had 80 participants, of whom 70 (87.5%) participants completed the t1 interview, and 57 (71.3%) participants completed the t2 interview (Figure 2). Regarding the secondary outcomes that were collected online, some missing data must also be reported. In total, 13 (16.3%) participants did not complete the t1 online questionnaires, and 30 (37.5%) participants did not complete the t2 online questionnaires. Therefore, the CC sample included 57 participants for the primary outcome and 50 participants for the secondary outcomes. Completion did not differ between the 2 groups for the primary (*t*_78_=–0.73; *P*=.23) and secondary (*t*_78_=–0.92; *P*=.18) outcomes. Of the participants in the VERP group, 4 participants did not take part in any therapy sessions. The remaining 36 participants attended an average of 5 (mean 5.31, SD 1.45) of the 6 sessions.

### CC Analyses

The results of the CC analyses are summarized in Table 3 for the primary outcome and in Table 4 for the secondary outcomes. For the primary outcome, there were no significant group differences (for the total scale and the 2 subscales of the Y-BOCS; all *P*>.05; η_p_^2^=0.003-0.014). This applies both to the change from t0 to t1 and to the change from t0 to t2.

**Table 3.** Primary outcomes at each assessment time for complete cases (CC) sample^a^.

	VERP^b^ group			CAU^c^ group			ANCOVA^j^ with respective baseline values as covariates							
	t0	t1	t2	t0	t1	t2	CC t0-t1			ITT^e^ t0-t1	CC t0-t2			ITT t0-t2
							*F* test (*df*=1,54)	*P* value	η_p_^2^	*P* value	*F* test (*df*=1,54)	*P* value	η_p_^2^	*P* value
Y-BOCS^f^ total	24.33 (6.03)	20.67 (7.01) ^g^	20.10 (7.22) [^g^	23.56 (5.50)	21.04 (6.07) ^h^	20.64 (6.11) ^h^	0.71	.40	0.013	.47	0.59	.45	0.011	.37
Y-BOCS obsessions	11.77 (3.16)	10.23 (3.81) ^h^	9.27 (3.78) ^g^	11.26 (2.92)	10.33 (3.16)	9.72 (3.31) ^i^	0.32	.57	0.006	.72	0.76	.39	0.014	.36
Y-BOCS compulsions	12.57 (3.22)	10.43 (3.40) ^g^	10.83 (3.82) ^g^	12.30 (3.12)	10.70 (3.41) ^g^	10.92 (3.32) ^h^	0.69	.41	0.013	.36	0.16	.70	0.003	.53

a ANCOVA with respective baseline values as covariates for CCs (n=57) and ITT (n=80) samples. ITT analyses were conducted using multiple imputation in SPSS. For these models, SPSS provides pooled significance tests (*P* values) but does not output pooled *F* statistics, degrees of freedom, or effect size estimates (eg, partial eta squared). Accordingly, only *P* values are reported for the ITT analyses. CC analyses are presented with full test statistics.

b VERP: virtual exposure with response prevention.

c CAU: care as usual.

d ANCOVA: analysis of covariance.

e ITT: intention-to-treat.

f Y-BOCS: Yale-Brown Obsessive-Compulsive Scale.

g *P*≤.001.

h *P*<.01.

i *P*<.05.

**Table 4.** Secondary outcomes at each assessment time for complete cases (CC) sample (n=50)^a^.

	VERP^b^ group			CAU^c^ group			ANCOVA^d^ with respective baseline values as covariates							
	t0	t1	t2	t0	t1	t2	CC t0-t1			ITT^e^ t0-t1	CC t0-t2			ITT t0-t2
							*F* test (*df*=1,47)	*P* value	η_p_^2^	*P* value	*F* test (*df*=1,47)	*P* value	η_p_^2^	*P* value
OCI-R^f^	33.74 (12.26)	30.41 (11.94)^g^	31.52 (12.03)	29.44 (11.67)	27.91 (13.01)	30.57 (15.45)	0.34	.56	0.007	.49	1.18	.28	0.024	.29
BDI-II^h^	19.93 (11.47)	18.07 (11.39)	20.15 (12.72)	20.83 (11.79)	19.78 (13.63)	19.04 (14.24)	0.20	.66	0.004	.86	0.47	.49	0.010	.55
GAD-7^i^	11.30 (5.14)	11.41 (5.73)	11.07 (4.84)	10.74 (4.63)	10.35 (6.01)	9.61 (5.66)	0.24	.62	0.005	.70	0.85	.36	0.018	.36
OBQ-44^j^	189.82 (49.38)	189.67 (50.44)	171.78 (51.62)^g^	187.44 (60.06)	180.09 (55.56)	167.52 (58.43)^k^	0.71	.40	0.015	.32	0.06	.81	0.001	.69
QoL^l^	2.81 (0.79)	2.93 (0.73)	2.85 (0.95)	2.87 (0.87)	3.17 (0.94)^g^	3.43(0.99)^g^	1.24	.27	0.026	.27	4.64	.04	0.090	.03

a ANCOVA with respective baseline values as covariates for CC (n=50) and ITT (n=80) samples. ITT analyses were conducted using multiple imputation in SPSS. For these models, SPSS provides pooled significance tests (*P* values) but does not output pooled *F* statistics, degrees of freedom, or effect size estimates (eg, partial eta square). Accordingly, only *P* values are reported for the ITT analyses. CC analyses are presented with full test statistics.

b VERP: virtual exposure with response prevention.

c CAU: care as usual.

d ANCOVA: analysis of covariance.

e ITT: intention-to-treat.

f OCI-R: Obsessive-Compulsive Inventory—Revised.

g *P*<.05.

h BDI-II: Beck Depression Inventory-II.

i GAD-7: Generalized Anxiety Disorder Scale-7.

j OBQ-44: Obsessive Beliefs Questionnaire-44.

k *P*<.001.

l Global item of the World Health Organization Quality of Life—BREF (quality of life).

For the within-group differences, both groups showed a significant improvement in the primary outcome from t0 to t1 (Y-BOCS total, VERP group: *t*_29_=3.95; *P*<.001; CAU group: *t*_26_=3.27; *P*=.002) and from t0 to t2 (Y-BOCS total, VERP group: *t*_29_=3.95; *P*<.001; CAU group: *t*_26_=2.95; *P*=.003).

With regard to the secondary outcomes, there was only 1 significant group difference in the change in quality of life (global item of the World Health Organization Quality of Life—BREF) from t0 to t2, favoring the CAU group (*F*_1,47_=4.64; *P*=.04; η_p_^2^=0.090). For all other secondary outcomes, there were no group differences from t0 to t1 or from t0 to t2 (all *P*>.05).

Significant within-group differences for secondary outcomes were only found for the change from t0 to t1 in the OCI-R (*t*_26_=2.14; *P*=.02) and from t0 to t1 in the OBQ-44 (*t*_26_=2.23; *P*=.02) in the VERP group. For the CAU group, within-group differences were found for the OBQ-44 between t0 to t2 (*t*_22_=3.60; *P*<.001) and for quality of life between t0 to t1 as well as between t0 and t2 (*t*_22_=–2.73; *P*=.006).

### ITT Analyses

The results of the ITT analyses were largely comparable to those of the CC analyses (Tables3  4). Again, only the improvement in quality of life showed a significant group difference from t0 to t2, favoring the CAU group (*P*=.03).

### Remission and Response

In total, 10 (17.5%) participants of the CC sample showed remission (Y-BOCS score ≤14 [60]) at t1, 6 (20%) patients of the VERP group, and 4 (14.8%) patients of the CAU group. At t2, 7 (23.3%) patients of the VERP group and 5 (18.5%) patients of the CAU group showed remission. In the VERP group, 5 of 30 (16.7%) participants can be regarded as responders (ie, reduction of >35% in primary outcome Y-BOCS) at t1 and 7 of 30 (23.3%) participants at t2. In the CAU group, 2 of 27 (7.4%) participants can be regarded as responders at t1, and 4 of 27 (14.8%) participants at t2.

### Sense of Presence

The mean value for the perceived sense of presence at post was 37.5 (SD 14.35) for the overall scale of IPQ, with the following mean values for the subscales: spatial presence 16.12 (SD 6.00), involvement 9.81 (SD 4.14), and experience realism 8.15 (SD 5.01). No significant correlations were present for any subscale of the IPQ nor for the total scale with the difference on the primary outcome and its subscales at each assessment time (*r*≤0.379; *P*≥.06). Sense of presence (total scale and subscales) did not differ between the 2 symptom subtypes (*P*≥.27).

### Exploratory Analyses Including OCD Subtype

**Figure 3.**
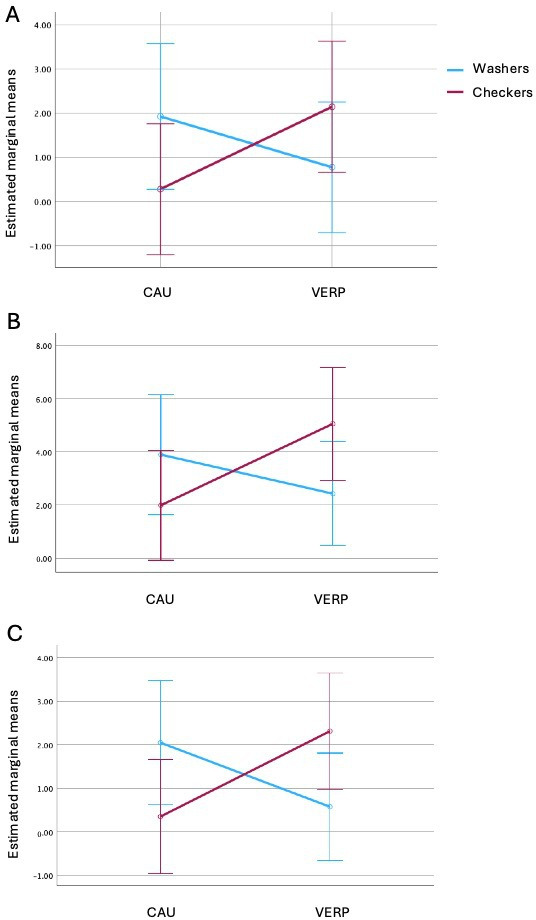
Significant interactions of exploratory 2-factor (group and type of obsessive-compulsive disorder) ANCOVA. Error bars: 95% CI. (A) Estimated marginal means of difference on subscale obsession of the Y-BOCS from baseline to post for the complete cases sample. Covariates appearing in the model were evaluated at Y-BOCS subscale obsession baseline=11.60. (B) Estimated marginal means of difference on the Y-BOCS total scale from baseline to post. Covariates appearing in the model were evaluated at Y-BOCS total scale baseline =24.36. (C) Estimated marginal means of difference on subscale obsession of the Y-BOCS from baseline to post for all available lost-to-follow-up data. Covariates appearing in the model were evaluated at Y-BOCS subscale baseline=11.60. ANCOVA: analysis of covariance; CAU: care as usual group; VERP: virtual exposure with response prevention; Y-BOCS: Yale-Brown Obsessive-Compulsive Scale.

When including OCD subtype (conOCD and checkOCD) as a fixed factor within a 2×2 factorial ANCOVA, we observed a trend toward an interaction effect between group and type of OCD for the change in obsessions (Y-BOCS subscale obsessions) from t0 to t1 (*F*_1,52_=3.91; *P*=.05, η_p_^2^=0.070) favoring the combination VERP group and patients with checkOCD (Figure 3). To further examine the trend-level interaction between OCD subtype and treatment group, we conducted simple-effects analyses. For patients with checkOCD, there was a statistical trend toward greater improvement in the VERP group compared to CAU alone (*F*_1,52_=3.16; *P*=.08). In contrast, among patients with conOCD, differences between VERP and CAU were not significant (*F*_1,52_=1.09; *P*=.30). Thus, the interaction pattern appears to be primarily driven by relatively greater symptom reduction among patients with checkOCD receiving CAU plus VERP, rather than by reduced improvement among patients with conOCD in CAU plus VERP. Additionally, we found a main effect for type of OCD for change in anxiety (GAD-7) from t0 to t2 (*F*_1,45_=4.11; *P*=.048; η_p_^2^=0.084), showing that patients with conOCD had a significantly greater decrease in anxiety compared to patients with checkOCD (mean difference for patients with conOCD 0.74, SD 3.84; mean difference for patients with checkOCD –0.41, SD 4.72).

To carry out the analysis with the largest possible sample, the same analysis was calculated with all available lost-to-t2 data for exploratory purposes; we additionally found a significant interaction effect between group and type of OCD for the change in OC symptoms (Y-BOCS total scale) from t0 to t1 (*F*_1,65_=4.64; *P*=.04; η_p_^2^=0.067) and a significant interaction effect between group and type of OCD for the change in obsessions (Y-BOCS subscale obsessions) from t0 to t1 (*F*_1,65_=6.66; *P*=.01; η_p_^2^=0.093), both favoring the combination VERP group and patients with checkOCD (Figure 3).

Descriptive data (means and SDs of Y-BOCS total and subscale scores at all 3 time points) for both subtypes are presented in MultimediaAppendices 1  2.

### Exploratory Moderation Analysis

Results of the exploratory moderation analysis are summarized in Table 5. Individuals in the VERP group who did not prematurely discontinue a previous treatment showed a significantly greater reduction on the Y-BOCS at t1 (*P*=.04) and t2 (*P*=.008) relative to the CAU group. In addition, individuals in the VERP group who scored high on compulsive checking (OCI-R subscale checking) showed a trend toward greater reduction on the primary outcome at post relative to the CAU group at t1 (OCI-R subscale checking: *P*=.05).

**Table 5.** Moderators for obsessive-compulsive symptom improvement (Y-BOCS^a^ total difference scores, means are centered), results of the complete cases sample.

	B (SE)	*t* test	*P* value	LLCI^c^	ULCI^d^	*P* for –1SD	*P* for 0	*P* for +1SD
Y-BOCS difference baseline to post								
Discontinuation of prior treatment	6.08 (2.83)	2.15	.04	0.40	11.75	.16	—^e^	.07
OCI-R^b^ subscale checking	0.75 (0.37)	2.00	.05	–0.002	1.50	.35	.31	.04
Y-BOCS difference baseline to follow-up								
Discontinuation of prior treatment	9.12 (3.31)	2.76	.008	2.49	15.76	.07	—	.03

a Y-BOCS: Yale-Brown Obsessive-Compulsive Scale.

b OCI-R: Obsessive-Compulsive Inventory—Revised.

c LLCI: lower level confidence interval.

d ULCI: upper level confidence interval.

e Not available.

### Subjective Appraisal

**Figure 4.**
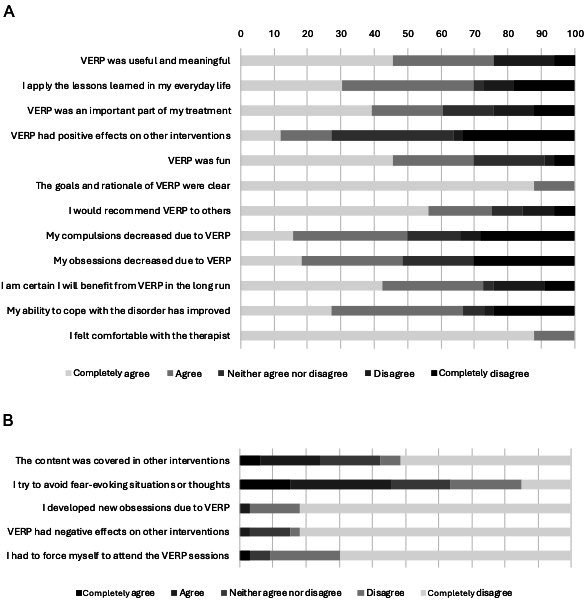
Subjective appraisal of VERP (n=33; n=32 for 2 items: My compulsions decreased due to VERP and I would recommend VERP to others). (A) Positive items and (B) reverse items. VERP: virtual exposure with response prevention.

Results of the subjective appraisal are summarized in Figure 4. The majority evaluated VERP as useful and meaningful (25/33, 75.8%) and that they would recommend it to others (24/32, 75%). The purpose of the intervention was clear to all participants (33/33, 100%), and all participants felt comfortable with the therapist (33/33, 100%). Half of the participants reported that their compulsions (16/32, 50%) and obsessions (16/33, 48.5%) decreased due to VERP. Only 1 (1/33, 3%) participant stated that VERP had a negative effect on other interventions, that they had to force themselves to attend the VERP sessions, and that they developed new obsessions due to VERP. Qualitative feedback on the intervention was positive. Most of the participants expressed their appreciation of and gratitude to the study therapists. One participant stated:

The threshold for entering exposure therapy has been significantly lowered through the use of virtual reality. In addition, you can playfully test and explore your own fears and compulsions in a safe environment and approach fearful objects with a safety net.

## Discussion

The aim of the study was to investigate the efficacy and acceptance of VERP as an add-on to CAU in a sample of patients with OCD and contamination-related (patients with conOCD) or checking-related (patients with checkOCD) compulsions and to identify possible moderators of treatment success.

### Main Findings

Within this randomized controlled trial, we did not find additional efficacy of VERP in the total sample of patients with OCD (patients with conOCD and patients with checkOCD) when measured after the 6-week intervention period (post) and 3 months after post. Both the VERP group and the CAU group improved in the primary outcome (OC symptom reduction). This result contrasts with findings from prior studies that have shown the efficacy of VERP in reducing OC symptoms [21,31]. One reason for this could be that the intervention period in the study by Javaherirenani et al [21] lasted for 12 weeks (ie, twice as long as in our study), which could have played a role in the more favorable outcomes observed in their study. In addition, the unusually high baseline Y-BOCS scores in their sample may have facilitated greater symptom reduction compared to our study. Furthermore, with only 1 session per week in our study, there is a lot of time between sessions, so old behavior patterns can quickly reappear. In line with this, there are highly effective concentrated therapeutic approaches in the treatment of OCD that aim for block-based treatment [61,62]. It is also conceivable that in VERP, the transfer from digital to everyday life is more difficult compared to ERP in vivo, meaning that generalization is less likely to take place. It is further possible that there was too little variance in our scenario (always the same therapy room with some repeated OC-relevant objects), as Craske et al [63] postulate that repeated practice in different contexts is important for the generalization of the knowledge acquired during ERP. In addition, compulsions are highly idiosyncratic, so our selection of objects may not have been truly relevant to every patient (some patients are more afraid of radiation or asbestos, eg, which we did not address in our study). Furthermore, in our study, a large proportion of the CAU group received outpatient psychotherapy during the intervention period, some with guided or unguided ERP in vivo, which may explain the improvement in the CAU group. Several technical and design-related factors may have also contributed to the absence of significant between-group effects. Locomotion in the virtual environments was implemented via teleportation, which—while reducing simulator sickness—may have disrupted spatial presence and attenuated emotional engagement compared to more naturalistic movement. In addition, the lack of haptic feedback (eg, touching a clean chair while viewing a contaminated stimulus) may have reduced perceptual realism and contributed to the moderate presence ratings observed.

Ecological validity may also have been limited in the checking scenario. Participants were instructed to switch devices off once and immediately leave the apartment, which may not have fully captured postdeparture doubt and urges to recheck—core features of checking compulsions. Together, these factors may have constrained anxiety induction and thereby reduced the observable impact of VERP on global OCD severity.

Importantly, the absence of even a trend-level difference between groups deserves further consideration. More fundamental explanations may include that the VERP component was not sufficiently potent to add therapeutic benefit beyond CAU, and that the heterogeneity of concurrent treatments (including therapist-guided ERP in both groups, albeit more frequently in the VERP group) likely diluted potential differences attributable to the experimental intervention. In addition, the limited sample size may have restricted our ability to detect subtle effects. Taken together, these factors suggest that the lack of group differences cannot be attributed solely to technical aspects of the intervention but may also reflect broader methodological and conceptual limitations.

Additionally, a low level of sense of presence could have played a role. However, only 1 of the 2 studies mentioned earlier assessed sense of presence [31], and its authors did not provide an interpretation of the scores assessed, so we cannot come to any conclusion regarding whether the observed effects might be due to a higher sense of presence. In our study, the reported level of sense of presence was only moderate (mean 37.5, SD 14.35; range 14-94). Although correlations between sense of presence and treatment outcome in our study did not reach statistical significance (*r*≤0.379; *P*≥.06), others found a higher sense of presence associated with higher experienced anxiety in VRET for anxiety disorders [33], which could also apply to OCD. The induction of distress or an unpleasant emotion such as anxiety or disgust is an essential component of ERP [5], and it is therefore possible that more positive results could have been achieved in our study if the experienced sense of presence had been higher (eg, if patients, especially patients with conOCD, could see their own hands in the VR environment [25,64]). Compared to in vivo ERP, the absence of genuine haptic contamination cues (eg, real dirt or moisture) may have limited the intensity of obsessive thoughts and emotional responses during VERP. This limitation, together with the only moderate levels of sense of presence reported in our study, might partly explain why VERP did not yield additional effects, in contrast to studies such as Javaherirenani et al [21], where patients physically touched dirty objects and repeated this as homework.

Another aspect might be that on an even more general level, the construct of sense of presence may not fully capture disorder-specific conditions of exposure in OCD, such as the threat of having actually contaminated oneself or overlooked something with catastrophic consequences. Since these fears often arise from patients’ own actions or omissions rather than visual input alone, VR-based exposure may elicit weaker emotional responses in OCD than in anxiety disorders.

Surprisingly, the only significant group difference found was an increase in quality of life from t0 to t2 in the CAU group compared to the VERP group, which was contrary to our expectations. A possible reason for this could be that participants who knew they were taking part in a trial may have experienced an improvement in their quality of life regardless of the actual treatment because they paid more attention to their behavior and symptoms and took better care of themselves overall. Another explanation could be that those participants of the CAU group who were not receiving treatment experienced a feeling of relief because they temporarily did not have to actively deal with their OCD.

### Subsample Analyses

If we take a closer look at the subsample analyses by content of OCD (patients with conOCD vs patients with checkOCD), we found a trend-level interaction between group and subtype for the change in obsessions from t0 to t1. This may indicate that the 2 subtypes responded differently to the intervention, with a possible tendency toward greater reduction in obsessions for patients with checkOCD. However, these findings should be interpreted with caution. Given the absence of an overall effect in the full sample, as well as the exploratory nature of these analyses, the results should be regarded as only preliminary indications that warrant replication in future studies with a priori hypotheses and larger samples. Moreover, the factorial validity of the Y-BOCS subscales themselves has been debated [65,66], further limiting the interpretability of subscore-based findings. From a clinical perspective, it is conceivable that differences in the predominant emotions associated with each subtype (fear in checkOCD and disgust in conOCD) may play a role in how patients engage with VR-based exposure. In our opinion, it would have seemed more plausible that patients with conOCD would show greater improvement, given that disgust can typically be elicited more directly by visual stimuli in VR, whereas anxiety in checkOCD is often tied to imagined future consequences (eg, forgetting to turn off the stove), which may be less readily activated in a virtual environment. At the same time, patients with conOCD might have engaged in more cognitive avoidance during exposure, which could have reduced the efficacy of VERP for this group—although this remains speculative, as our measures did not allow us to test this assumption. It is also possible that the abovementioned reasons for the general lack of effects apply even more to patients with conOCD (high idiosyncrasy, too little variance in the scenario, unsuitable stimuli, etc).

We also found a main effect for subtype, with patients with conOCD showing a greater decrease in anxiety from t0 to t2. A possible explanation could be that washing compulsions are often more directly linked to immediate anxiety reduction, whereas checking compulsions are often tied to future-oriented threats. This might make patients with conOCD more responsive to interventions aimed at anxiety reduction.

### Exploratory Moderation Analysis

The findings of the exploratory moderation analysis provide deeper insight into the factors that may influence the effectiveness of adding VERP to CAU. Individuals in the VERP group who scored high on compulsive checking showed a trend toward greater reductions in the primary outcome than those in the CAU group at t1. This suggests that CAU plus VERP may be particularly effective for individuals with compulsive checking behavior, indicating that this subtype of OCD may benefit more from VERP, which is also implied by the subgroup analyses. Comparable studies examining the efficacy of VERP have so far only included patients with washing compulsions [21,31], so we cannot draw a comparison here.

Individuals in the VERP group who had not previously discontinued treatment prematurely showed a significantly greater reduction in OC symptoms at both t1 and t2 compared to those in the CAU group, indicating that patients with a history of consistent treatment may respond better to CAU plus VERP or other forms of treatment, highlighting the importance of adherence to treatment protocols for therapeutic success [67]. Consistency in treatment reflects a level of engagement and commitment to the therapeutic process. It indicates that the patient has built a degree of resilience and is willing to work through the discomfort that is often part of exposure-based therapies such as VERP. This consistency allows for greater opportunities for learning and adaptation, which are crucial factors in reducing OC symptoms.

### Subjective Acceptance

The subjective evaluation of VERP was positive overall, particularly with regard to the usefulness, purpose, fun, and applicability of the intervention as well as the therapeutic relationship, but it was rather moderate with regard to the reduction of OC symptoms. However, the results of the subjective evaluation indicate that VERP may be better accepted than ERP in vivo [14], although no direct comparison is possible based on the present study design.

### Strengths and Limitations

Strengths of the study were the inclusion of an active control group, the verification of diagnoses using standardized clinical interviews by trained and blinded raters, and the assessment of self-rating psychopathology questionnaires as well as the collection of follow-up data. At the same time, several limitations should be noted.

First, the intervention period was rather short, consisting of only 6 weekly sessions, of which the first 2 were primarily preparatory and only approximately 4 sessions involved active exposure exercises. This represents a substantially lower treatment “dose” than standard ERP protocols, which typically include a higher number of exposure sessions over a longer time frame. Given the chronic nature of OCD in this sample (mean illness duration of 18.04, SD 14.22 years), it is questionable whether this limited exposure intensity was theoretically sufficient to induce measurable changes in global OCD severity as assessed by the Y-BOCS. Consequently, the relatively brief intervention duration and limited number of active exposure sessions may have been a primary contributor to the small and nonsignificant effect sizes observed, particularly with respect to overall symptom reduction.

Second, although the study used a randomized controlled design, the control condition was treatment as CAU in outpatient care and not an active comparator (eg, ERP in vivo); therefore, conclusions about the relative efficacy of VERP compared to standard ERP in vivo cannot be drawn. In addition, a substantial proportion of participants in the CAU group received concurrent outpatient psychotherapy, and more than half were exposed to guided or unguided ERP as part of their usual care. This overlap with established evidence-based interventions likely reduced the contrast between groups and may have diluted potential add-on effects of VERP, thereby contributing to the absence of significant between-group differences.

Third, the sample was restricted to 2 symptom subtypes (conOCD and checkOCD). The findings may thus not generalize to the broader spectrum of OC presentations. Participants were assigned to subtypes based on their predominant symptom dimension, although OCD symptoms frequently co-occur across dimensions. This binary classification may therefore oversimplify the heterogeneous nature of OCD and may have resulted in misclassification, potentially attenuating or obscuring differential intervention effects between subgroups.

In addition, although diagnoses were verified using the Mini-International Neuropsychiatric Interview and OCD symptom severity was assessed with the gold-standard semistructured Y-BOCS interview, several secondary outcomes (eg, OCI-R and BDI-II) relied on self-report and were assessed without blinding. These measures may thus have been susceptible to response biases, including demand characteristics in the intervention group or resentful demoralization in the control group, which may have influenced symptom ratings independently of true treatment effects. In addition, sample size within subgroups was modest, which limited the power to detect smaller effects and to explore interaction effects across different clinical characteristics.

Fourth, dropout and prior treatment history influenced treatment response, as indicated by the moderation analysis. This points to selection effects and highlights the need to replicate the findings in a larger, more heterogeneous sample. Although missing data were handled using multiple imputation under a missing-at-random assumption, no formal sensitivity analyses under nonmissing-at-random assumptions were conducted. If participants who discontinued follow-up assessments experienced poorer outcomes, treatment effects may have been overestimated.

Fifth, longer-term effects beyond the 3-month follow-up were not assessed. Therefore, the durability of the effects, particularly the differential effects for conOCD and checkOCD, remains to be determined. A further limitation concerns the sample size estimation. Because empirical data on VERP in OCD were scarce at the time of study planning, our power analysis was based on effect sizes reported for VRET in anxiety disorders. Given that previous VERP trials in OCD have not shown effects as large as those in anxiety disorders, this assumption may have been overly optimistic. As a result, our study might have been underpowered to detect more modest effects of VERP in OCD.

Moreover, the relatively small sample size may limit the reliability of the trend-level interaction effects observed in subgroup analyses, which should therefore be interpreted with caution.

Another limitation concerns the exploratory nature of the analyses of Y-BOCS subscales. These measures are highly interrelated, and multiple testing increases the risk of type I error. Therefore, the results on subscales need to be interpreted cautiously, and we note that the study was primarily powered to detect differences in the Y-BOCS total score. Exploratory moderation analyses based on single-item measures should also be interpreted with caution and warrant replication using validated instruments and prespecified hypotheses.

Furthermore, medication use was assessed only at baseline, and no monitoring or management of psychotropic medication occurred during the study. Consequently, changes in medication during the intervention period could not be controlled and may have confounded symptom change, potentially masking or mimicking intervention effects.

Additionally, recruitment partly involved contacting participants from previous studies, which may have introduced a selection bias toward individuals with more chronic or treatment-refractory OCD, potentially contributing to the high chronicity of the sample and the limited treatment response observed.

Finally, the add-on nature of the trial limits the interpretation of our findings. Because both groups received CAU and only the intervention group received additional VERP sessions, the results demonstrate the incremental benefit of VERP but do not allow conclusions about the efficacy of VERP as a stand-alone treatment or in direct comparison with established ERP. We deliberately chose this design because VERP is still in an early stage of evaluation, and testing it as an adjunct to CAU offered a pragmatic and ethically appropriate first step that mirrors real-world clinical practice. Future studies using alternative designs (eg, noninferiority trials or head-to-head comparisons) will be needed to clarify the relative effectiveness of VERP within the broader spectrum of OCD interventions.

### Recommendations

Further research should examine whether VERP offers greater efficacy than standard treatments with extended intervention periods and advanced virtual environments, as well as in comparison to in vivo ERP. Studies should also investigate how the sense of presence relates to treatment outcomes and how it can be increased (possibly by using mixed reality approaches) and assess VERP’s effectiveness for other OCD types.

### Conclusions

The study revealed that CAU plus VERP did not show a significant effect in reducing OC symptoms in the whole sample of patients with conOCD and patients with checkOCD after a 6-week intervention and a 3-month follow-up. However, exploratory subsample analyses provided preliminary indications that adding VERP to CAU might be associated with greater reductions in obsessions among patients with checkOCD and with greater reductions in anxiety among patients with conOCD. Similarly, patients in the VERP group who reported checking compulsions and who had not previously discontinued treatment prematurely showed signals of greater improvement in OC symptoms at posttreatment and follow-up compared to those in the CAU group. Given the exploratory nature and methodological limitations of these analyses, these findings should be interpreted with caution and considered hypothesis-generating. While VERP was generally well accepted by participants, several factors could play a role in the current nonsignificant treatment effects.

## Supplementary material

10.2196/79326 Multimedia Appendix 1 Yale-Brown Obsessive-Compulsive Scale interview.

10.2196/79326 Multimedia Appendix 2 Descriptives (means and SDs) of the Yale-Brown Obsessive-Compulsive Scale total score and its subscales (obsession and compulsions) at all 3 assessment times for the 2 obsessive-compulsive disorder subtypes.

10.2196/79326 Checklist 1 CONSORT checklist.

## Abbreviations

ANCOVA: analysis of covariance
BDI-II: Beck Depression Inventory-II
CAU: care as usual
CBT: cognitive behavioral therapy
CC: complete case
checkOCD: checking obsessive-compulsive disorder
conOCD: contamination obsessive-compulsive disorder
CONSORT: Consolidated Standards of Reporting Trials
ERP: exposure with response prevention
GAD-7: Generalized Anxiety Disorder Scale-7
IPQ: Igroup Presence Questionnaire
ITT: intention-to-treat
OBQ-44: Obsessive Beliefs Questionnaire-44
OC: obsessive-compulsive
OCD: obsessive-compulsive disorder
OCI-R: Obsessive-Compulsive Inventory—Revised
VERP: virtual exposure with response prevention
VR: virtual reality
VRET: exposure therapy in virtual reality
Y-BOCS: Yale-Brown Obsessive-Compulsive Scale
