# Supplementary material for: Virtual Exposure With Response Prevention for Obsessive-Compulsive Disorder: Randomized Controlled Trial
Source: J Med Internet Res. 2026 May 27;28:e79326. doi: 10.2196/79326 (PMC13215575; doi:10.2196/79326)
Supplement: Multimedia Appendix 1 [file jmir-v28-e79326-s001.pdf]

Y-BOCS – UKE Version

Yale-Brown Obsessive Compulsive Scale (1986 und 1989, dt. Übersetzung Büttner-Westphal, 1991)

Version inhaltsgleich zum Original, Hinweise an Rater:innen aber teils erweitert oder gekürzt (Änderungen farblich markiert)

Y-BOCS-Gesamtscore (= Summe Items 1-10 ohne 1b und 6b): ☐ t0 ☐ t1 ☐ t2 ☐ t3 ☐

Patient:innen-Code: ______________ Interviewer:in: _____________________ Datum: _____________

1. Einführung

Ehe Sie zu den Fragen übergehen, definieren Sie der Patientin / dem Patienten „Zwangsgedanken" und „Zwangshandlungen" folgendermaßen[1]:

„**Zwangsgedanken** sind störende Ideen, Gedanken, Bilder oder Impulse, die Ihnen wiederholt in den Sinn kommen. Sie können sich anscheinend gegen Ihren Willen aufdrängen. Sie können Ihnen zuwider sein oder sie können Ihnen als sinnlos und persönlichkeitsfremd erscheinen."

Patient:in nach eigenen Beispielen fragen und diese ggf. notieren:

Notizen: ____

„**Zwangshandlungen** sind auf der anderen Seite Verhaltensweisen oder Handlungen, zu deren Ausführung Sie sich gedrängt fühlen, obwohl Sie diese möglicherweise als sinnlos oder übertrieben ansehen. Manchmal kann es sein, dass Sie versuchen, ihnen zu widerstehen, was sich jedoch als schwierig herausstellen mag. Möglicherweise erleben Sie Angst, die nicht abnimmt, bevor die Handlung abgeschlossen ist. Zu den Zwangshandlungen zählen auch alle gedanklichen Handlungen. Also auch wenn Sie ein Verhalten nur in Gedanken ausüben, betrachten wir das als mentale Handlung. Bitte berücksichtigen Sie das bei Ihren Antworten im Folgenden."

Patient:in nach eigenen Beispielen fragen und diese ggf. notieren:

Notizen: ____

2. Checkliste[2]

Geben Sie alle Zwänge an, die genannt werden, aber heben Sie die Hauptsymptome mit einem zusätzlichen „H“ hervor. (Interviewer:in muss klären, ob angegebene Verhaltensweisen tatsächlich Symptome einer Zwangsstörung und keiner anderen Störung darstellen. Die in der Checkliste mit einem „*“ markierten Items können nicht eindeutig der Zwangsstörung bzw. einer anderen Störung zugeordnet werden.)

Zwangsgedanken

Aggressive Zwangsgedanken[3]

	gegenwärtig	früher
Befürchtungen, sich selbst zu verletzen	__	__
Befürchtungen, andere zu verletzen	__	__
Gewalttätige oder schreckenerregende Vorstellungen	__	__
Befürchtungen, obszöne Gedanken oder Beleidigungen laut von sich zu geben	__	__
Befürchtungen, etwas Peinliches zu tun*	__	__
Befürchtungen, aufgrund unkontrollierbarer Impulse zu handeln (z. B. auf eine nahestehende Person mit dem Messer einzustechen)	__	__
Befürchtungen, einen Diebstahl zu begehen	__	__
Befürchtungen, andere zu verletzen, weil man nicht aufmerksam genug ist (z. B. Befürchtung, jemand unbemerkt mit dem Auto angefahren zu haben und dann durch das Weiterfahren Fahrerflucht begangen zu haben)	__	__
Befürchtungen, dafür verantwortlich zu sein, dass etwas (anderes als oben bereits angegebenes) Schreckliches passiert (z.B. Feuer, Einbruch)	__	__
Sonstiges	__	__

Zwangsgedanken, die sich auf Verschmutzung beziehen[4]

	gegenwärtig	früher
Sorgen über oder Ekel in Bezug auf körperliche Ausscheidungen (z. B. Urin, Fäzes, Speichel)	__	__
Sorgen über Schmutz oder Keime	__	__
Exzessive Sorgen über Verunreinigungen in der Umgebung (z. B. Asbest, Strahlen, giftige Abfallstoffe)	__	__
Exzessive Sorgen über Dinge im Haushalt (z. B. Reinigungsmittel, Lösungsmittel)	__	__
Exzessive Sorgen über bestimmte Tiere (z. B. Insekten)	__	__
Sich durch klebrige Substanzen oder andere Rückstände beunruhigt fühlen	__	__
Sorgen darüber, aufgrund von Verschmutzung oder Verkeimung krank zu werden	__	__
Sorgen darüber, andere dadurch krank zu machen, dass man Keime oder Verschmutzungen weitergibt (aggressive Komponente)	__	__
Keine Sorgen über Konsequenzen einer Verschmutzung oder Verkeimung außer derjenigen, welche Gefühle dadurch ausgelöst werden[6]	__	__
Sonstiges	__	__

Zwangsgedanken mit sexuellem Inhalt[4]

	gegenwärtig	früher
Verbotene oder tabuisierte[7] sexuelle Gedanken, Bilder oder Impulse	__	__
Auf Kinder oder Inzest bezogene Inhalte	__	__
Auf Homosexualität bezogene Inhalte*[8]	__	__
Sexuelles Verhalten anderen gegenüber (mit aggressiver Komponente)*	__	__
Sonstiges	__	__

Zwangsgedanken, die sich auf das Sammeln und das Aufbewahren von Gegenständen beziehen[4]

	gegenwärtig	früher
(Abzugrenzen gegenüber Hobbies und Beschäftigung mit Objekten, die finanziell wertvoll sind oder einen besonderen persönlichen Erinnerungswert haben)	__	__

Zwangsgedanken mit religiösen und solchen Inhalten, die ein schlechtes Gewissen erzeugen[4]

	gegenwärtig	früher
Befürchtung, Gotteslästerungen zu begehen	__	__
Übermäßige Beschäftigung mit Fragen der Moral und richtigen oder falschen Handlungen	__	__

Zwangsgedanken, die sich auf Symmetrie oder Genauigkeit beziehen[4]

	gegenwärtig	früher
In Verbindung mit magischem Denken (z. B. Befürchtung, dass die Mutter einen Unfall haben wird, wenn Dinge nicht am richtigen Platz stehen)	__	__
Nicht in Verbindung mit magischem Denken	__	__

Zwangsgedanken in Bezug auf den eigenen Körper[4]

	gegenwärtig	früher
Besorgnis über Missempfindungen oder Krankheiten	__	__
Exzessive Sorgen hinsichtlich bestimmter Körperteile oder Besonderheiten des Aussehens (z. B. Dysmorphophobie)*	__	__

Sonstiges	__	__

Verschiedene Zwangsgedanken[3]

Drang, Dinge wissen oder erinnern zu müssen	__	__
Furcht, bestimmte Dinge zu sagen	__	__
Furcht, nicht das Richtige zu sagen	__	__
Furcht, Dinge zu verlieren	__	__
Sich aufdrängende (nicht gewaltbezogene) Bilder	__	__
Sich aufdrängende unsinnige Geräusche, Wörter oder Musik	__	__
Sich belästigt fühlen durch bestimmte Töne oder Geräusche*	__	__

	gegenwärtig	früher
Glücks-/Unglückszahlen	__	__
Farben mit bestimmter Bedeutung	__	__
Abergläubische Befürchtungen	__	__
Sonstiges	__	__

Zwangshandlungen

Reinigungs-Wasch-Zwänge[4]

Exzessives oder ritualisiertes Händewaschen	__	__
Exzessives oder ritualisiertes Duschen, Baden, Zähneputzen oder andere Körperpflege	__	__
Beschäftigung mit der Reinigung von Haushalts- oder anderen Gegenständen	__	__
Andere Maßnahmen, um Kontakt mit Verschmutzungen zu vermeiden oder zu beseitigen	__	__
Sonstiges	__	__

Kontrollzwänge[5]

Kontrollieren von Schlössern, Herd, Elektrogeräten usw.	__	__
Kontrollieren, ob man andere Menschen verletzt hat / Vorsichtsmaßnahmen, dass dies nicht passieren wird	__	__
Kontrollieren, ob man sich selbst verletzt hat / Vorsichtsmaßnahmen, dass dies nicht passieren wird	__	__
Kontrollieren, ob nichts Schreckliches passiert ist oder passieren wird	__	__
Kontrollieren, ob man keinen Fehler gemacht hat	__	__
Kontrollieren in Verbindung mit Befürchtungen hinsichtlich des eigenen Körpers	__	__

Sonstiges	__	__

Wiederholungszwänge[4]

	gegenwärtig	früher
Mehrmaliges Lesen oder Schreiben	__	__
Notwendigkeit, Routineaktivitäten zu wiederholen (z. B. durch die	__	__
Tür hinein- und hinausgehen, sich auf den Stuhl hinsetzen und aufstehen)	__	__
Sonstiges	__	__

**Zählzwänge**	__	__
**Ordnungszwänge**	__	__

Sammel- und Aufbewahrungszwänge

	__	__

(Abgrenzen gegenüber Hobbies und Beschäftigung mit Objekten, die finanziell wertvoll sind oder einen besonderen persönlichen Erinnerungswert haben), z. B. sorgfältiges Durchlesen von Reklamesendungen, Aufeinanderstapeln alter Zeitungen, Durchwühlen von Abfall, Sammeln von nutzlosen Gegenständen

Verschiedene Zwangshandlungen[3]

Gedankliche Rituale (außer Kontrollieren, Zählen)	__	__
Exzessives Erstellen von Listen über Alltagsdinge	__	__
Drang zu reden, fragen oder bekennen[9]	__	__
Drang, Dinge anzufassen, anzutippen oder zu reiben*	__	__
Rituale, die Blinzeln oder Anstarren beinhalten*	__	__
Maßnahmen (nicht Kontrollieren) zur Verhinderung von Selbstverletzungen / Verletzungen anderer Personen / schrecklichen Konsequenzen	__	__
Ritualisiertes Essverhalten*	__	__
Abergläubische Verhaltensweisen	__	__
Trichotillomanie*, Dermatillomanie oder Onychophagie	__	__
Andere selbstschädigende oder selbstverletzende Verhaltensweisen*	__	__
Sonstiges	__	__

3. Liste der Zielsymptome der individuellen Zwänge

Zwangsgedanken

1.____

2.____

3.____

Zwangshandlungen

1.____

2.____

3.____

Vermeidung

1.____

2.____

3.____

4. Yale-Brown Obsessive Compulsive Scale

„Ich werde Ihnen jetzt verschiedene Fragen über Ihre Zwangsgedanken stellen.“[10] (Beziehen Sie sich dabei vor allem auf die Hauptsymptome der Patientin / des Patienten.)

1) Zeitaufwand für die Beschäftigung mit Zwangsgedanken
„Wie viel Zeit nimmt die Beschäftigung mit Zwangsgedanken[11] in Anspruch?“ (wenn kurze, wiederkehrende, sich aufdrängende Gedanken, nach Häufigkeit und Anzahl betroffener Stunden pro Tag erfragen + „Wie häufig treten die Zwangsgedanken auf?“. Ich-syntone und rationale aber übertriebene Grübeleien hiervon ausschließen.)

0 = keine Beschäftigung mit Zwangsgedanken
1 = leichtes (weniger als eine Stunde pro Tag) oder gelegentliches Aufdrängen von Gedanken
2 = mäßiges (ein bis drei Stunden täglich) oder häufiges Aufdrängen von Gedanken, wobei die meisten Stunden des Tages nicht betroffen sind
3 = starkes (mehr als drei und bis zu acht Stunden täglich) oder sehr häufiges Aufdrängen von Gedanken und während der meisten Stunden des Tages
4 = extremes (mehr als acht Stunden täglich) oder fast ständiges Aufdrängen von Gedanken

1b) Intervalle, in denen keine Zwangsgedanken auftreten
für den Gesamtscore nicht berücksichtigen!
„Welche ist im Durchschnitt die längste zusammenhängende Anzahl von Stunden pro Tag, in der Sie völlig frei von Zwangsgedanken sind?“ (Falls nötig, fragen Sie; „Was ist der längste Zeitabschnitt, über den keine Zwangsgedanken auftreten?“)

0 = keine Symptome
1 = langes symptomfreies Intervall, mehr als acht zusammenhängende Stunden Symptomfreiheit pro Tag
2 = mäßig langes symptomfreies Intervall, mehr als drei und bis zu acht zusammenhängende Stunden Symptomfreiheit pro Tag
3 = kurzes symptomfreies Intervall, ein bis drei zusammenhängende Stunden Symptomfreiheit pro Tag
4 = extrem kurzes symptomfreies Intervall, weniger als eine zusammenhängende Stunde Symptomfreiheit pro Tag

2) Beeinträchtigungen durch Zwangsgedanken
„Wie stark werden Sie durch Ihre Zwangsgedanken im Umgang mit anderen Menschen oder bei der Arbeit beeinträchtigt? Gibt es irgendetwas, das Sie deswegen nicht tun?“ (Falls Patient:in zurzeit nicht berufstätig ist, ist festzustellen, inwieweit bei Berufstätigkeit Beeinträchtigung vorliegen würde.)

0 = keine Beeinträchtigung
1 = leichte oder wenig Beeinträchtigung im Umgang mit anderen oder bei der Arbeit, Aufgaben können aber noch voll erfüllt werden
2 = mäßige aber schon deutliche Beeinträchtigung im Umgang mit anderen bzw. am Arbeitsplatz, Aufgaben können jedoch noch ausreichend erfüllt werden
3 = starke Beeinträchtigung im Umgang mit anderen bzw. am Arbeitsplatz, Aufgaben können nicht mehr ausreichend erfüllt werden
4 = extreme Beeinträchtigung im Alltagsleben

3) Leidensdruck in Verbindung mit Zwangsgedanken
„Wie stark werden Sie durch Ihre Zwangsgedanken geplagt?“ (Meistens Korrelation von Leidensdruck und Ausmaß begleitender Ängste. Andere empfinden Zwänge eher als „störend“, ohne dass Angst vorliegt. Nur Angst berücksichtigen, die durch Zwangsgedanken hervorgerufen wird, keine generalisierte oder durch andere Symptome verursachte Angst.)

0 = gar nicht
1 = leicht, nicht allzu störend
2 = mäßig, störend aber noch zu bewältigen
3 = stark, sehr störend
4 = extremer, nahezu kontinuierlicher und lähmender Leidensdruck

4) Widerstand gegen Zwangsgedanken
„Wie stark bemühen Sie sich, den Zwangsgedanken zu widerstehen? Wie oft versuchen Sie, diese Gedanken nicht zu beachten bzw. sich abzulenken, wenn sie sich Ihnen aufdrängen?“ (Nur Bemühung um Widerstand einschätzen, nicht das Gelingen. Item bemisst gesunde Anteile des Patienten / der Patientin i. S. v. Anstrengung, die er / sie gegen Zwangsgedanken unternimmt, ohne Zwangs- oder Vermeidungshandlungen auszuführen. „Aktive“ und „passive“ Formen des Widerstands berücksichtigen, z.B. Anwendung verhaltenstherapeutischer Techniken wie passive Opposition[12] zählen als Sonderformen von Widerstand. Auch dysfunktionalen Widerstand einbeziehen, solange er gezeigt wird.)

0 = strengt sich an, immer zu widerstehen bzw. die Symptome sind so minimal, dass es nicht nötig ist, dagegen Widerstand zu leisten
1 = versucht fast immer zu widerstehen
2 = macht einige Anstrengungen zu widerstehen
3 = lässt alle Zwangsgedanken zu ohne den Versuch, diese zu kontrollieren, tut dies jedoch mit einigem Widerstreben
4 = lässt die Zwangsgedanken vollständig und bereitwillig zu

5) Grad der Kontrolle über die Zwangsgedanken
„Wieviel Kontrolle haben Sie über Ihre Zwangsgedanken? Wie erfolgreich sind Sie dabei, Ihre Zwangsgedanken zu beenden bzw. sich davon abzulenken? Können Sie Ihre Zwangsgedanken einfach übergehen?“ (enger mit Ausprägungsgrad der Zwangsgedanken und Funktionalität, also dem langfristigen Erfolg des Widerstandes verbunden.)

0 = völlige Kontrolle
1 = starke Kontrolle, die normalerweise mit einigem Aufwand und Konzentration die Zwangsgedanken beendet oder davon ablenkt
2 = mäßige Kontrolle, die manchmal die Zwangsgedanken beendet oder davon ablenkt
3 = wenig Kontrolle, Patient:in ist selten erfolgreich im Beenden der Zwangsgedanken beendet, kann die Aufmerksamkeit nur unter Schwierigkeiten ablenken
4 = keine Kontrolle, Zwangsgedanken werden als völlig unkontrollierbar erfahren. Patient:in ist selten fähig, die Gedanken auch nur kurzfristig zu ändern

Teilscore: Zwangsgedanken (Summe 1—5 ohne 1b)

„Das waren die Fragen zu den Zwangsgedanken. Nun wird es ausschließlich um Ihre (mentalen) Zwangshandlungen gehen, also die Rituale, die sie tatsächlich oder in Gedanken ausführen. Bitte beziehen Sie sich in Ihren Antworten nur darauf.“

6) Dauer der Ausführung von Zwangshandlungen
„Wie viel Zeit nimmt die Ausführung von Zwangshandlungen in Anspruch?“[13] (bei vielen Ritualen fragen Sie: „Wieviel länger brauchen Sie wegen Ihrer Rituale im Vergleich zu den meisten Menschen, um Routinetätigkeiten auszuführen?“. Bei kurzen, wiederkehrenden Verhaltensweisen nach Häufigkeit dieser fragen, um Zeitdauer abzuschätzen. Auftretenshäufigkeit nicht Wiederholungshäufigkeit abfragen. Fragen Sie „Wie oft führen Sie Zwangshandlungen aus?“)

0 = kein Zeitaufwand
1 = leichte oder gelegentliche Ausführung von Zwangshandlungen (weniger als eine Stunde pro Tag)
2 = mäßige oder häufige Ausführung von Zwangshandlungen (eine bis drei Stunden täglich)
3 = starke oder sehr häufige Ausführung von Zwangshandlungen (mehr als drei und bis zu acht Stunden täglich)
4 = extreme oder fast durchgängige Ausführung von Zwangshandlungen (mehr als acht Stunden täglich)

6b) Intervalle, in denen keine Zwangshandlungen auftreten
für den Gesamtscore nicht berücksichtigen
„Welche ist im Durchschnitt die längste zusammenhängende Anzahl von Stunden pro Tag, in der Sie völlig frei von Zwangshandlungen sind?“ (falls nötig, fragen Sie: „Was ist der längste Zeitabschnitt, über den keine Zwangshandlungen auftreten?“)

0 = keine Symptome
1 = langes symptomfreies Intervall, mehr als acht zusammenhängende Stunden Symptomfreiheit pro Tag
2 = mäßig langes symptomfreies Intervall, mehr als drei und bis zu acht zusammenhängende Stunden Symptomfreiheit pro Tag
3 = kurzes symptomfreies Intervall, eine bis drei zusammenhängende Stunden Symptomfreiheit pro Tag
4 = extrem kurzes symptomfreies Intervall, weniger als eine zusammenhängende Stunde Symptomfreiheit pro Tag

7) Beeinträchtigung durch Zwangshandlungen
„Wie stark werden Sie durch Ihre Zwangshandlungen im Umgang mit anderen Menschen oder bei der Arbeit beeinträchtigt? Gibt es irgendetwas, das Sie deswegen nicht tun?“ (Wenn Patient:in zurzeit nicht berufstätig ist, ist festzustellen, inwieweit bei Berufstätigkeit eine Beeinträchtigung vorliegen würde.)

0 = keine Beeinträchtigung
1 = leichte oder wenig Beeinträchtigung im Umgang mit anderen Menschen oder am Arbeitsplatz, dabei insgesamt keine Funktionsverminderung
2 = mäßige aber schon deutliche Beeinträchtigung im Umgang mit anderen Menschen oder am Arbeitsplatz, jedoch noch zu bewältigen
3 = starke Beeinträchtigung im Umgang mit anderen Menschen bzw. am Arbeitsplatz
4 = extreme Behinderung in der täglichen Lebensführung

8) Leidensdruck in Verbindung mit Zwangshandlungen

„Wie ginge es Ihnen, wenn Sie an der Ausführung Ihrer Zwangshandlungen gehindert werden würden?“ (Pause) „Wie ängstlich oder beunruhigt würden Sie dann werden?“[14] (meint plötzliche Unterbrechung, ohne Rückversicherung. Meist wird Angst / Unruhe / Missempfindung durch Ausführung reduziert. Wenn laut klinischem Eindruck Angst gerade durch Verhinderung der Zwangshandlung reduziert wird, fragen Sie: „Wie ängstlich / beunruhigt werden Sie, während Sie Zwangshandlungen ausführen oder zu einem für Sie befriedigenden Abschluss bringen?“)

0 = gar nicht ängstlich / beunruhigt
1 = wenig, nur leicht ängstlich / beunruhigt, wenn Zwangshandlungen verhindert werden oder nur leichte Angst / Unruhe / Missempfindungen während ihrer Ausführung
2 = mäßig, Patient:in berichtet, dass Angst / Unruhe / Missempfindungen ansteigen, aber zugleich noch zu bewältigen sein würden, wenn Zwangshandlungen verhindert werden würden bzw., dass sie während der Ausführung von Zwangshandlungen zwar ansteigen, aber noch zu bewältigen sein würden
3 = starke, vorherrschende und als sehr störend empfundene Zunahme der Angst / Unruhe / Missempfindungen, wenn die Zwangshandlungen unterbrochen werden würden oder vorherrschende und als sehr störend empfundene Zunahme derselben während der Ausführung von Zwangshandlungen
4 = extreme, behindernde Angst / Unruhe / Missempfindungen, die entweder durch Interventionen, die versuchen das Zwangsverhalten zu verhindern, provoziert werden oder die auch während der Durchführung einer Zwangshandlung entstehen würden

9) Widerstand gegen die Zwangshandlungen
„Wie stark bemühen Sie sich, den Zwangshandlungen zu widerstehen?“ (Nur versuchten Widerstand einschätzen, nicht das Gelingen. Item bemisst gesunde Anteile und Funktionstüchtigkeit des Patienten / der Patientin i. S. v. Anstrengung.)

0 = bemüht sich immer zu widerstehen, oder die Symptome sind so minimal, dass es nicht nötig ist, Widerstand zu leisten
1 = versucht meistens zu widerstehen
2 = macht einige Anstrengungen zu widerstehen
3 = führt fast alle Zwangshandlungen aus ohne den Versuch, diese zu kontrollieren, tut dies aber mit einigem Widerstreben
4 = führt alle Zwangshandlungen vollständig und bereitwillig aus

10) Ausmaß der Kontrolle über Zwangshandlungen
„Wie stark ist der Drang, die Zwangshandlungen auszuführen? (Pause) „Wieviel Kontrollen haben Sie über die Zwangshandlungen?“ (enger mit Ausprägungsgrad der Zwangshandlungen verbunden.)

0 = völlige Kontrolle
1 = starke Kontrolle, Patient:in empfindet den Drang, die Handlung auszuführen, ist jedoch gewöhnlich in der Lage, willkürliche Kontrolle darüber auszuüben
2 = mäßige Kontrolle, starker Drang zur Ausführung der Handlung, Patient:in kann sie nur unter Schwierigkeiten kontrollieren
3 = wenig Kontrolle, sehr starker Drang zur Ausführung der Handlung, Handlung muss bis zum Ende ausgeführt werden, Patient:in kann sie nur unter Schwierigkeiten hinauszögern
4 = keine Kontrolle, Drang zur Ausführung der Handlung wird als völlig unfreiwillig und unkontrollierbar empfunden, Patient:in ist selten fähig, die Handlung auch nur kurzfristig zu verzögern

Teilscore: Zwangshandlungen (Summe 6—10 ohne 6b)

„Die weiteren Fragen beziehen sich sowohl auf Ihre Zwangsgedanken wie Ihre Zwangshandlungen. Einige beziehen sich auch auf damit verbundene Probleme.“ (Diese Items dienen vorerst der Hypothesenbildung und werden nicht in den Gesamtwert der Y-BOCS einbezogen, sie können aber hilfreich bei der Gesamtbeurteilung dieser Symptome sein.)

11) Einsicht in die Zwangsgedanken und -handlungen
„Glauben Sie, dass Ihre Befürchtungen oder Verhaltensweisen berechtigt sind? „(Pause) „Was meinen Sie würde passieren, wenn Sie die Zwangshandlung(en) nicht ausführen? Sind Sie überzeugt davon, dass tatsächlich etwas passieren würde?“ (Einsicht von Patient:in außerhalb ihrer Zwänge in Sinnlosigkeit und Übertriebenheit der Zwangshandlung(en) einschätzen, ausgehend von während des Interviews geäußerten Überzeugungen oder Annahmen.)

0 = hervorragende Einsicht, völlig rational
1 = gute Einsicht; Patient:in gibt bereitwillig die Absurdität oder Übertriebenheit seiner / ihrer (Zwangs-)Gedanken oder Verhaltensweisen zu, scheint aber nicht völlig überzeugt davon zu sein, dass es neben der Angst nicht doch noch etwas gibt, über das man sich Sorgen machen müsste (d.h. hat anhaltende Zweifel)
2 = mäßige Einsicht; Patient:in gibt widerwillig zu, dass seine / ihre Gedanken oder Verhaltensweisen unsinnig oder übertrieben scheinen, ist jedoch unschlüssig. Möglicherweise

hat er / sie einige unrealistische Ängste, jedoch keine dahinterstehenden festen Überzeugungen.
3 = wenig Einsicht; Patient:in behauptet, das seine / ihre Gedanken oder Verhaltensweisen nicht unsinnig oder übertrieben sind (d h. es gibt überwertige Ideen)
4 = keine Kontrolle, wahnhaft, Patient:in ist entschieden überzeugt, dass Befürchtungen und Verhaltensweisen berechtigt sind. Er / Sie reagiert nicht auf Gegenbeweise.

12) Vermeidung
„Vermeiden Sie, irgendwelche Dinge zu tun, irgendwo hinzugehen oder aber mit jemandem zusammen zu sein, weil Zwangsgedanken auftreten könnten oder weil sie befürchten, Zwangshandlungen ausführen zu müssen?" Bei ‚Ja': „Wie sehr vermeiden Sie?" (Ausmaß einschätzen, in dem Patient:in vorsätzlich versucht, Dinge zu vermeiden. Zwangshandlungen zur Kontaktvermeidung sind nicht gemeint.)

0 = kein vorsätzliches Vermeidungsverhalten
1 = leicht: minimales Vermeidungsverhalten
2 = mäßig: etwas Vermeidungsverhalten deutlich zu erkennen
3 = stark: häufiges Vermeidungsverhalten, Vermeidung steht im Vordergrund
4 = extrem: sehr ausgeprägtes Vermeidungsverhalten, Patient:in bemüht sich in jeder Hinsicht zu vermeiden, dass Symptome ausgelöst werden

13) Ausmaß von Entscheidungsschwierigkeiten
„Haben Sie Probleme, Entscheidungen über unbedeutende Dinge zu treffen, über die andere Leute nicht zweimal nachdenken würden? Z. B. darüber, welche Kleidung Sie morgens anziehen oder welche Müslimarke Sie kaufen wollen?" (meint Entscheidungsschwierigkeiten ohne Zusammenhang zu Zwangsgrübeleien. Ambivalenz in Bezug auf tatsächlich als schwierig einzustufende Entscheidungssituation ausschließen.)

0 = keine Entscheidungsschwierigkeiten
1 = leicht: einige Probleme, Entscheidungen über geringfügige Dinge zu treffen
2 = mäßig: Patient:in berichtet offen über erhebliche Schwierigkeiten, Entscheidungen zu treffen in Bereichen, in denen andere Menschen nicht zweimal nachdenken müssten
3 = stark: fortlaufendes Abwägen von Pro und Contra bei Nebensächlichkeiten
4 = extrem: unfähig, irgendwelche Entscheidungen zu treffen, mit entsprechender Behinderung

14) Übertriebenes Verantwortungsgefühl
„Fühlen Sie sich sehr verantwortlich für die Konsequenzen Ihrer Handlungen? Geben Sie sich selbst die Schuld für die Konsequenzen von Ereignissen, die nicht völlig unter Ihrer Kontrolle sind?" (Dies ist zu trennen von normalen Verantwortungsgefühlen, Gefühlen der Wertlosigkeit und pathologischen Schuldgefühlen.)

0 = kein übertriebenes Verantwortungsgefühl
1 = leicht: wird nur auf Anfrage erwähnt
2 = mäßig: entsprechende Gedanken werden aber spontan geäußert, sind eindeutig vorhanden; Patient:in erlebt ein beträchtliches Ausmaß an übertriebenem Verantwortungsgefühl für Ereignisse, die außerhalb der tatsächlichen Kontrollen liegen
3 = stark: entsprechende Gedanken sind vorherrschend und eindringlich; Patient:in ist zutiefst besorgt, dass er / sie für Ereignisse verantwortlich ist, die außerhalb der tatsächlichen Kontrolle liegen, entwickelt Schuldgefühle in übertriebener und nahezu irrationaler Weise
4 = extrem: wahnhaft anmutendes Verantwortungsgefühl (z. B. Verantwortungsgefühl bei Erdbeben in 3000 Meilen Entfernung, weil man seine Zwangshandlung nicht ausgeführt hat.)

15) Anhaltende Langsamkeit / Trägheit
„Haben Sie Schwierigkeiten, Aufgaben anzufangen oder zu beenden? Dauern viele Ihrer Routinetätigkeiten länger, als sie eigentlich dauern sollten?" (psychomotorische Hemmung im Rahmen einer Depression nicht werten. Zeitaufwand bei Abwicklung von Routineaktivitäten auch werten, wenn spezifische, zugehörige Zwangsgedanken nicht identifiziert werden können.)

0 = keine anhaltende Langsamkeit / Trägheit
1 = leicht: gelegentliche Verzögerung beim Beginnen oder Beenden von Routineaktivitäten
2 = mäßig: häufiges Ausdehnen von Routineaktivitäten, wobei Handlungen normalerweise noch abgeschlossen werden: häufiges Zuspätkommen
3 = stark: anhaltende und auffällige Schwierigkeiten beim Beginnen oder Beenden von Routinetätigkeiten; regelmäßiges Zuspätkommen
4 = extrem: Patient:in ist nicht in der Lage, ohne fremde Hilfe Routinetätigkeiten zu beginnen oder zu beenden.

16) Pathologisches Zweifeln
„Haben Sie am Ende einer Handlung Zweifel, ob Sie sie richtig ausgeführt haben? Zweifeln Sie daran, ob Sie sie überhaupt getan haben? Haben Sie den Eindruck, dass Sie Ihren eigenen Wahrnehmungen nicht trauen, wenn Sie Routineaktivitäten durchführen – z. B. im Hinblick auf das, was Sie sehen, hören oder berühren?"

0 = keine Zweifel
1 = leicht: wird nur auf Anfrage erwähnt, leichte pathologische Zweifel. Die angegebenen Beispiele könnten noch im Normalbereich liegen
2 = mäßig: Gedanken werden spontan zugegeben, sie sind eindeutig vorhanden und werden zumindest in einigen der Handlungen der Patientin / des Patienten deutlich; Patient:in wird von deutlichen pathologischen Zweifeln geplagt; diese haben einige Rückwirkungen auf das Verhalten, sind aber noch zu bewältigen
3 = stark: Unsicherheit in Bezug auf Wahrnehmung oder Gedächtnis steht im Vordergrund; pathologisches Zweifeln beeinträchtigt häufig das Verhalten
4 = extrem: Unsicherheit in Bezug auf Wahrnehmung ist durchgängig vorhanden; pathologisches Zweifeln beeinträchtigt nachhaltig nahezu alle Aktivitäten; schwere Beeinträchtigung (z. B. „Mein Verstand traut dem nicht, was meine Augen sehen“)

Die letzten drei Items sind von Interviewer:in ohne direkte Frage an Patient:in einzuschätzen. Items 17 und 18 beziehen sich auf den Schweregrad der Zwangsstörung der Patientin / des Patienten. Die Interviewerin / der Interviewer sollte daher die globale Funktionsfähigkeit und nicht nur die Schwere der Zwangssymptomatik berücksichtigen.

17) Gesamtschweregrad
(Berücksichtigen Sie berichteten Leidensdruck, beobachtete Symptome, berichtete Funktionsbeeinträchtigung im Alltagsleben, also im Interview erhaltene Informationen.)

0 = keine Störung
1 = Störung geringfügig, zweifelhaft, vorübergehend; keine Funktionsbeeinträchtigung
2 = leichte Symptome; geringe Funktionsbeeinträchtigung
3 = mäßige Symptome; Funktionsfähigkeit nur noch mit Anstrengung aufrechterhalten
4 = mäßige bis schwere Symptome; eingeschränkte Funktionstüchtigkeit
5 = schwere Symptome; Patient:in im Alltag überwiegend auf fremde Hilfe angewiesen
6 = extrem schwere Symptome; völlige Funktionsunfähigkeit

18) Gesamtverbesserung
(Schätzen Sie bitte die Gesamtverbesserung seit der ersten Beurteilung an, unabhängig davon, ob dies nach Ihrer Meinung auf eine (z. B. medikamentöse) Behandlung zurückzuführen ist oder nicht.)

0 = sehr viel schlechter
1 = viel schlechter
2 = geringfügig schlechter
3 = keine Veränderung
4 = geringfügig besser
5 = viel besser
6 = sehr viel besser

19) Reliabilität
(Gesamtreliabilität der erhaltenen Informationen einschätzen. Möglicherweise beeinträchtigende Faktoren: Kooperation und Kommunikationsfähigkeit der Patientin / des Patienten. Art und Schweregrad der Zwangssymptome können Konzentration, Aufmerksamkeit oder Spontanität beeinträchtigen.)

0 = ausgezeichnet, kein Anlass, den Wahrheitsgehalt der Informationen anzuzweifeln
1 = gut, Faktor(en) vorhanden, der / die möglicherweise die Reliabilität ungünstig beeinflusst / beeinflussen
2 = befriedigend, Faktor(en) vorhanden, der / die definitiv die Reliabilität verringert / verringern
3 = unzureichend, sehr niedrige Reliabilität

Legende zu Veränderungen hinsichtlich der Originalversion + Durchführungshinweise und –hilfen, bitte immer in die Testungen mitnehmen:

blau gefärbt: ergänzt

grün gefärbt: Position geändert

rot gefärbt: gekürzt im Vergleich zu Original, Sinn wurde beibehalten

[1] Durchführungshinweis: dazu sagen „Damit wir beide unter Zwangsgedanken und –handlungen dasselbe verstehen, möchte ich Ihnen dazu Definitionen vorlesen und Sie um eigene Beispiele dazu bitten.“.

[2] Durchführungshinweis: dazusagen „gegenwärtig“ bezieht sich auf die letzten sieben Tage, während „früher“ alles davor meint + Durchführungshilfe zur Fragenformulierung: „Kennen Sie … gegenwärtig oder von früher?“ + Durchführungshinweis: Überschrift der Zwangskategorien nicht explizit nennen.

[3] Bei dieser Item-Sammlung alle Items abfragen.

[4] Durchführungshinweis: Pro Themenblock fragen, ob der für Patient:in Relevanz hat, sonst zum nächsten Block übergehen. Hier reicht ein Beispiel-Item.

[5] Durchführungshinweis: Pro Themenblock fragen, ob der für Patient:in Relevanz hat, sonst zum nächsten Block übergehen. Hier zur Klarheit verschiedene Beispiel-Items aus der Kategorie nennen.

[6] Durchführungshinweis: meint Emotionen wie z. B. Ekel oder Anspannung.

[7] Im Original steht „perverse“ statt „tabuisierte Inhalte“.

[8] Durchführungshinweis: dazusagen „Es geht um unerwünschte, sich aufdrängende Inhalte, die können auch von der eigenen sexuellen Orientierung abweichen“.

[9] Durchführungshinweis: meint z.B. sich zur Richtigkeit des eigenen Handelns bei Autoritätsperson rückversichern.

[10] Durchführungshinweis: Nochmal Hinweis mit individuellem Beispiel auf Unterschied Gedanken / gedankliche Handlungen und Rituale.

[11] Durchführungshinweis: gemeint sind alle genannten Zwangsgedanken in der Summe + Durchführungshilfe: den typischen Tagesablauf der Teilnehmenden durchgehen, ab wann und in wie vielen Lebensbereichen Zwangsgedanken auftreten.

[12] Durchführungshinweis: wenn Patient:in eine Technik nutzt, nachfragen, woher er / sie diese kennt -> alle Techniken sind als Versuch/Bemühung zu werten, Nachfragen soll nur helfen, auch passive Formen des Widerstands als solchen zu erkennen.

[13] Durchführungshinweis: Alle genannten Zwangshandlungen inkl. verdeckter Handlungen wie gedanklichem Neutralisieren, Rationalisieren etc..

[14] Durchführungshinweis: sollte der klinische Eindruck von Einschätzung von Patient:in abweichen: imaginativ nachhaken (z. B. „Stellen Sie sich bitte einmal bildlich vor, wie Sie … (nicht) tun. Wie geht es Ihnen mit dieser Vorstellung?“).

WICHTIG: Der Text der ersten Seite des Originals wurde stark gekürzt, für Einarbeitung neuer Tester:innen empfehlen wir unbedingt, dass sie / er sich auch die Originalversion durchliest, da die vorliegende Version wichtige Hintergrundinformationen zur Testgüte und -durchführung ausspart.
