# Supplementary material for: Virtual Exposure With Response Prevention for Obsessive-Compulsive Disorder: Randomized Controlled Trial
Source: J Med Internet Res. 2026 May 27;28:e79326. doi: 10.2196/79326 (PMC13215575; doi:10.2196/79326)
Supplement: Multimedia Appendix 2 [file jmir-v28-e79326-s002.docx]

**Table S1.** Descriptives (means and SDs) of the Y-BOCS total score and its subscales (obsession and compulsions) at all 3 assessment times for the 2 OCD subtypes.

|  | Patients with checkOCD^b^ | | | Patients with conOCD^c^ | | |
| --- | --- | --- | --- | --- | --- | --- |
|  | t0 | t1 | t2 | t0 | t1 | t2 |
| Y-BOCS^a^ total scale | 24.40 (6.06) | 21.07 (6.78) | 20.52 (7.00) | 23.48 (5.45) | 20.59 (6.36) | 20.17 (6.41) |
| Y-BOCS obsessions | 11.60 (3.32) | 10.37 (3.58) | 9.38 (3.80) | 11.44 (2.74) | 10.19 (3.45) | 9.59 (3.30) |
| Y-BOCS compulsions | 12.80 (3.26) | 10.70 (3.48) | 11.13 (3.56) | 12.04 (3.03) | 10.41 (3.23) | 10.58 (3.60) |

^a^ Yale-Brown Obsessive-Compulsive Scale

^b^ checking OCD

^c^ contamination OCD
